# Structure, function, and assembly of PSI in thylakoid membranes of vascular plants

**DOI:** 10.1093/plcell/koae169

**Published:** 2024-06-07

**Authors:** David Rolo, Mark A Schöttler, Omar Sandoval-Ibáñez, Ralph Bock

**Affiliations:** Max Planck Institute of Molecular Plant Physiology, Am Mühlenberg 1, 14476 Potsdam-Golm, Germany; Max Planck Institute of Molecular Plant Physiology, Am Mühlenberg 1, 14476 Potsdam-Golm, Germany; Max Planck Institute of Molecular Plant Physiology, Am Mühlenberg 1, 14476 Potsdam-Golm, Germany; Max Planck Institute of Molecular Plant Physiology, Am Mühlenberg 1, 14476 Potsdam-Golm, Germany

## Abstract

The photosynthetic apparatus is formed by thylakoid membrane-embedded multiprotein complexes that carry out linear electron transport in oxygenic photosynthesis. The machinery is largely conserved from cyanobacteria to land plants, and structure and function of the protein complexes involved are relatively well studied. By contrast, how the machinery is assembled in thylakoid membranes remains poorly understood. The complexes participating in photosynthetic electron transfer are composed of many proteins, pigments, and redox-active cofactors, whose temporally and spatially highly coordinated incorporation is essential to build functional mature complexes. Several proteins, jointly referred to as assembly factors, engage in the biogenesis of these complexes to bring the components together in a step-wise manner, in the right order and time. In this review, we focus on the biogenesis of the terminal protein supercomplex of the photosynthetic electron transport chain, PSI, in vascular plants. We summarize our current knowledge of the assembly process and the factors involved and describe the challenges associated with resolving the assembly pathway in molecular detail.

## Introduction

PSI is a large membrane protein complex composed of protein subunits, pigments such as chlorophylls and carotenoids, and cofactors. Together with PSII, the cytochrome *b*_6_*f* complex, and the plastid ATP synthase (ATPase), it forms the thylakoid membrane-embedded machinery conducting the light reactions of photosynthesis. The photosynthetic apparatus is largely conserved from oxygenic photosynthetic prokaryotes to eukaryotes. Despite their structural and functional similarities (including evolutionarily related reaction centers, and the utilization of chlorophylls for light harvesting and electron transport), the 2 photosystems are distinct entities with numerous unique properties. The terminal acceptor of PSI at the stromal side represents a crucial point for the routing of electrons toward linear or cyclic electron flow (LEF vs CEF), which is particularly important for the regulation of photosynthesis (Suorsa 2015). The photo-energized reaction center of PSI, P_700_*, is the strongest biological reductant known (Amunts and Nelson 2009). PSI is also considered the most efficient biological energy converter (Nelson and Ben-Shem 2004; Amunts and Nelson 2009). One electron is energized per almost every single photon absorbed, meaning that the maximum quantum efficiency of the complex, that is, the number of electrons driven in the photosynthetic electron transport chain (ETC) per photon, is close to 100%, while it is only 80% to 90% in PSII-LHCII (Wientjes et al. 2013; Caffarri et al. 2014).

In photosynthetic eukaryotes, photosynthesis takes place in specialized organelles, the chloroplasts. In seed plants, chloroplast biogenesis occurs in young developing leaves and involves the differentiation of proplastids into chloroplasts (Mechela et al. 2019; Choi et al. 2021). During this process, the photosynthetic apparatus is co-assembled with lipids into thylakoid membranes inside the chloroplast (Rast et al. 2015; Mechela et al. 2019). This is a critical process for plant survival, as photosynthesis provides the only carbon source after the resources stored in the seed have been used up. Although the structure and function of the photosynthetic apparatus are relatively well known, the mechanisms of its assembly in thylakoid membranes are incompletely understood, especially regarding PSI. Nonetheless, substantial progress has been made in the past 3 decades toward deciphering the PSI assembly process, not the least due to the elucidation of the complex structure and the characterization of PSI-deficient mutants in model species of cyanobacteria, green algae, and land plants (reviewed by Schöttler et al. 2011; Yang et al. 2015). PSI assembly models, with relatively low resolution compared with PSII assembly models, have been proposed (Ozawa et al. 2010; Wittenberg et al. 2017), and a non-exhaustive list of auxiliary proteins, collectively referred to as PSI assembly factors, was reported to mediate PSI subunit incorporation during complex biogenesis (Schöttler et al. 2011; Yang et al. 2015; Nellaepalli et al. 2021). Here, we review our current understanding of PSI assembly with a focus on vascular plants, especially the model angiosperm species Arabidopsis (*Arabidopsis thaliana*), tobacco (*Nicotiana tabacum*), and maize (*Zea mays*). Where appropriate, we also integrate findings from cyanobacteria and green algae, especially Chlamydomonas (*Chlamydomonas reinhardtii*). After summarizing the present knowledge of PSI structure and function, we describe recent insights in the step-wise assembly of PSI, the roles that assembly factors play in this process, and the coevolution of the PSI structure with its assembly machinery. Finally, we highlight challenges associated with modelling PSI assembly and resolving the process in molecular detail.

## PSI and photosynthesis

### The photosynthetic apparatus in thylakoid membranes

**Figure 1.**
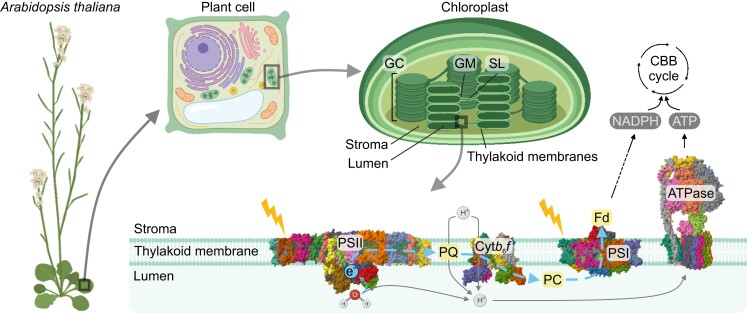
**PSI is embedded in the thylakoid membranes, compartmentalized in the chloroplast of plants and algae.** Land plants, including the model plant Arabidopsis, compartmentalize photosynthesis in specialized organelles, the chloroplasts. The first phase of photosynthesis occurs in the thylakoid membranes that separate the chloroplast stroma from the aqueous phase in the thylakoid lumen. Thylakoid membranes are organized in grana cores (GC), grana margins (GM), and stroma lamellae (SL). In linear electron flow, PSII, Cyt*b*_6_*f*, and PSI complexes participate in the electron (e^−^) transport to produce NADPH and generate a proton (H^+^) gradient across the thylakoid membrane to produce ATP by the ATPase. Light-harvesting complexes associated with both photosystems facilitate their photo-excitation to drive electron transport. NADPH and ATP fuel the Calvin-Benson-Bassham (CBB) cycle taking place in the stroma, and other anabolic processes. Structures of the PSII dimer in pea (*Pisum sativum*; Protein Data Bank Identifier (PDB ID): 5XNL; Su et al. 2017), the cytochrome *b*_6_*f* dimer in spinach (*Spinacia oleracea*; PDB ID: 7ZYV; Sarewicz et al. 2023), the PSI monomer in Arabidopsis (PDB ID: 8J7A; Wu et al. 2023), and the ATPase in spinach (PDB ID: 6FKF; Hahn et al. 2018) were retrieved from the Research Collaboratory for Structural Bioinformatics (RCSB) PDB website (https://www.rcsb.org/; Berman et al. 2000) and visualized in Mol* Viewer (https://molstar.org/viewer/; Sehnal et al. 2021). All other cartoons were retrieved from BioRender (https://biorender.com/). Abbreviations: Fd, ferredoxin; PC, plastocyanin; PQ, plastoquinone.

In eukaryotes, photosynthesis is compartmentalized in a specialized differentiation form of plastids, the chloroplast (Fig. 1). According to the endosymbiotic theory, chloroplasts originate from the symbiosis of a cyanobacterium with an ancestral eukaryotic host cell (Zimorski et al. 2014). In leaves of angiosperms, each mesophyll cell can contain up to approximately 200 chloroplasts (Kubínová et al. 2014), with number and size depending on internal and external factors (Xiong et al. 2017). In chloroplasts, 4 large membrane-embedded protein complexes, PSII and its associated light harvesting complex II (PSII-LHCII), Cyt*b*_6_*f*, PSI and the associated LHCI (PSI-LHCI), and the plastid ATPase form the core of the photosynthetic apparatus. The 4 complexes are embedded in the thylakoid membranes, which form a network surrounded by the stroma, and contain an inner matrix, the thylakoid lumen (Fig. 1). Thylakoid membranes are a dynamic network composed of stroma lamellae (non-appressed membranes) and stacked membranes that form the grana cores (Fig. 1; Li et al. 2020). The less densely stacked periphery of the grana core is called grana margins. Thylakoids are formed by a lipid bilayer largely saturated with proteins, which occupy up to 80% of the thylakoid membrane area (Kirchhoff et al. 2002, 2008). The photosynthetic protein complexes are not evenly distributed in the thylakoid membranes and, instead, are confined to different regions, a phenomenon known as lateral heterogeneity (Albertsson 2001; Anderson 2012). The Cyt*b*_6_*f* is evenly distributed in grana cores and stroma lamellae, with higher accumulation in grana margins, while the PSII-LHCII supercomplex is enriched in grana cores (Rantala et al. 2020). By contrast, PSI-LHCI is enriched in stroma lamellae (also found in grana margins), and the ATPase is enriched in grana margins (also present in stroma lamellae). Both PSI-LHCI and ATPase are sterically excluded from the grana cores due to their stromal protrusions (Fig. 1; Nevo et al. 2012; Rantala et al. 2020). Together with the mobile redox carriers plastoquinone (PQ) and plastocyanin, which transfer electrons from PSII to Cyt*b*_6_*f* and from Cyt*b*_6_*f* to PSI, respectively, these complexes are essential for the “light reactions” of photosynthesis, in which chemical energy in the form of ATP and reducing power in the form of reduced NADPH are produced (Fig. 1; Stirbet et al. 2020). The products of the light reactions are consumed by the “dark reactions,” or, more precisely, the carbon reactions of photosynthesis that take place in the stroma and lead to carbon assimilation by the conversion of carbon dioxide (CO_2_) into sugar via the Calvin–Benson–Bassham cycle (Fig. 1; Bassham et al. 1950) and its central CO_2_-fixing enzyme RuBisCO.

### Photosynthetic electron transport from PSII to PSI

In the light reactions of photosynthesis (Fig. 1), sunlight is captured by the antennae associated with both photosystems to provide the energy that drives the photosynthetic ETC to generate NADPH in the stroma (Fig. 1; Govindjee and Björn 2017). In parallel, fueled by the ETC, generation of a proton motive force (pmf) across the thylakoid membrane allows the ATPase to convert stromal ADP and orthophosphate (P_i_) into ATP (Junge and Nelson 2015).

To enable the utilization of light energy, both photosystems contain pigments, chlorophylls (and carotenoids), which are distributed in LHCs and complex cores (Nelson and Yocum 2006). PSII-LHCII, Cyt*b*_6_*f*, and PSI-LHCI protein subunits additionally bind diverse redox-active cofactors, including iron–sulfur [Fe–S] clusters, hemes, and quinones (Johnson 2016).

Light harvested by LHCII provides the energy to excite the PSII reaction center P_680_ into P_680_*. Electrons are then shuttled to the lipophilic carrier PQ at the PSII acceptor side, reducing PQ to plastoquinol (PQH). Photo-oxidized P_680_^+^ is reduced again by the oxygen-evolving complex (OEC) at the lumenal side of the dimeric PSII core (Fig. 1). The OEC is composed of proteins that bind a manganese [Mn_4_CaO_5_] cluster and, after 4 subsequent oxidation steps by P_680_^+^, catalyzes the oxidation of 2 molecules of H_2_O, releasing 4 H^+^ into the lumen and 1 molecule of dioxygen (O_2_) as a by-product.

The dimeric Cyt*b*_6_*f* complex contains the iron-sulfur [Fe_2_–S_2_] clusters and hemes involved in the reoxidation of PQH to PQ, and electrons circulate within Cyt*b*_6_*f* in a process known as the Q-cycle. During the Q-cycle, stromal protons are translocated to the thylakoid lumen, contributing to the formation of the pmf across the thylakoid membrane. Electrons are then transported from Cyt*b*_6_*f* to the PSI reaction center via the lumenal soluble carrier protein plastocyanin. Plastocyanin is reoxidized by P_700_^+^, the photo-oxidized chlorophyll-a dimer in the PSI reaction center core, after transfer of an electron from the reaction center to the stromal side of PSI to reduce ferredoxin. Consequently, reduced ferredoxin is reoxidized by the ferredoxin-NADP^+^ reductase (FNR), which in turn converts NADP^+^ into NADPH in the final step of LEF, thus providing reducing energy for the Calvin–Benson–Bassham cycle and other biosynthetic pathways. Alternatively, ferredoxin can directly reduce substrates in the nitrogen (nitrite reductase and glutamine: 2-oxoglutarate aminotransferase) and sulfur (sulfite reductase) assimilation pathways and participate in the redox regulation of chloroplast metabolism via the thioredoxin system (reviewed, e.g. by Hanke and Mulo 2013).

Electrons from ferredoxin can re-enter the electron transport chain in CEF, which uses different pathways to redirect electrons from the PSI-LHCI acceptor side to Cyt*b*_6_*f*, thus increasing the pmf without concomitant production of NADPH. Currently, 3 different pathways of CEF are discussed in angiosperms, but their quantitative contributions to total CEF and pmf formation remain controversial. CEF is believed to comprise an antimycin A-sensitive pathway possibly involving PGR5 and PGRL1 (Munekage et al. 2002; DalCorso et al. 2008; Hertle et al. 2013), and the plastid NADH-dehydrogenase-like (NDH) complex pathway (Joët et al. 2001; Peltier et al. 2016). Due to the low abundance of the NDH complex in most C_3_ plants (McKenzie et al. 2020), this pathway seems unlikely to catalyze the major route of CEF. Moreover, as in *pgrl1* mutants, high rates of CEF can still be observed; it has been proposed that direct reduction of Cyt*b*_6_*f*, for example, via an FNR directly bound to its stromal surface (Zhang et al. 2001; Szymańska et al. 2011), may be the dominant pathway of CEF (Nawrocki et al. 2019).

Besides increasing ATP production relative to NADPH synthesis, CEF may also protect PSI-LHCI against excessive electron transfer from PSII-LHCII. In addition to downregulation of ATPase activity (reviewed by Schöttler et al. 2015), CEF is a further mechanism promoting thylakoid lumen acidification. Lumenal pH values below 6.5 increasingly slow down PQH reoxidation by Cyt*b*_6_*f* in a process called “photosynthetic control” (Takizawa et al. 2007). As a result, the high-potential chain from cytochrome *f* to the chlorophyll-a dimer in the PSI reaction center P_700_ becomes increasingly oxidized. Because P_700_^+^ is a rather moderate oxidant and a safe quencher of excess excitation energy, this efficiently protects PSI against oxidative damage (Ort 2001). Only when photosynthetic control is insufficient to keep P_700_^+^ oxidized and electrons start to accumulate at the PSI acceptor side (see below), massive production of reactive oxygen species (ROS), especially the superoxide anion radical O_2_^−^, can occur. When superoxide is formed within the PSI reaction center itself, structural damage to PSI ensues and PSI photoinhibition can occur (reviewed by Krieger-Liszkay and Shimakawa 2022). Although photosynthetic control results in decreased LEF and over-reduction of the PSII acceptor side and the PQH pool, generation of ROS at PSII is efficiently minimized by the dissipation of excess excitation energy as heat via nonphotochemical quenching in the PSII antenna bed, a defense mechanism also induced by lumen acidification (Takizawa et al. 2007; Tikkanen et al. 2014). In addition, rapid repair mechanisms to replace photodamaged PSII complexes have evolved (reviewed by Su et al. 2024).

## PSI structure and function

**Figure 2.**
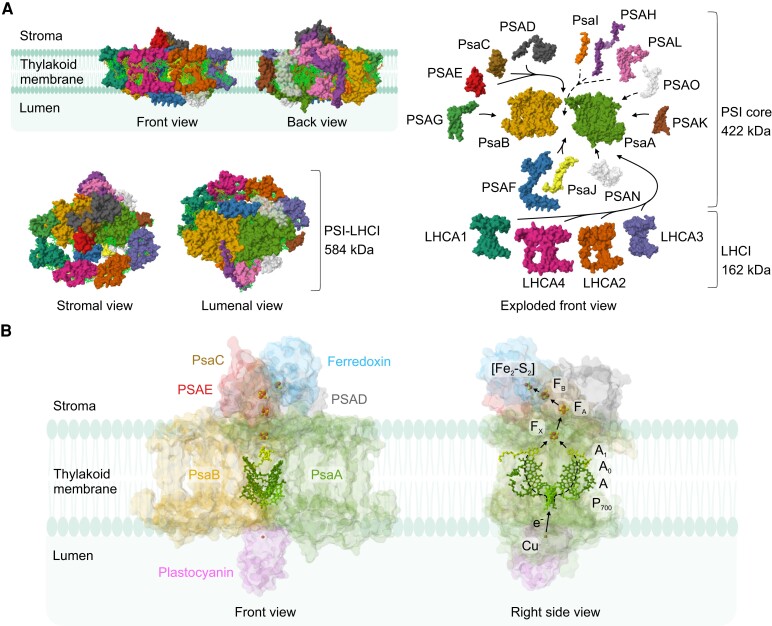
**PSI-LHCI supercomplex structure and function in land plants. A)** PSI-LHCI structure. PSI-LHCI complex subunits are represented by their structures from Arabidopsis (PDB ID: 8J7A; Wu et al. 2023). Only chlorophyll (light green) and carotenoids (orange) are shown among PSI-LHCI ligands in the front, back, stromal, and lumenal views. For clarity, cofactors were omitted from the exploded view. The “front” view (with the LHCI belt in the front) and “back” view of the complex are arbitrary. The subunits PSAO and PSAN were not resolved in this structure and, therefore, their structures and positions are displayed according to the PSI-LHCI-LHCII structure from maize (*Zea mays*; PDB ID: 5ZJI; Pan et al. 2018). **B)** PSI acts as a plastocyanin-ferredoxin oxidoreductase via a series of cofactors. Electrons are transferred from the copper (Cu)-containing carrier plastocyanin in the lumen to the primary electron donor P_700_, which translocates electrons to the [Fe_4_–S_4_] cluster F_x_ via the A-branch (left dashed line) and B-branch (right dashed line) in PsaA and PsaB, respectively, which contain both chlorophyll A and A_0_ and phylloquinone A1. Then, electrons are transferred to the PsaC-containing [Fe_4_–S_4_] clusters F_A_ and subsequently F_B_. Finally, the [Fe_2_–S_2_] cluster-containing ferredoxin, docked on the stromal ridge, is reduced. The subunits PsaA-E, plastocyanin, ferredoxin, and the cofactors involved in electron transport were resolved in the structure of the PSI-LHCI-plastocyanin-ferredoxin supercomplex from pea (*Pisum sativum*; PDB ID: 6YEZ; Caspy et al. 2020). All structures were retrieved from RCSB PDB website (https://www.rcsb.org/; Berman et al. 2000) and visualized in Mol* Viewer (https://molstar.org/viewer/; Sehnal et al. 2021). The cartoon of the membrane was retrieved from BioRender (https://biorender.com/).

In angiosperms, the PSI core is composed of 14 subunits and LHCI is composed of 4 subunits (Table 1 and Fig. 2A). Together, they form the PSI-LHCI complex, which amounts to a molecular mass of nearly 600 kDa. As a legacy of their cyanobacterial origin, chloroplasts possess their own genome (Zimorski et al. 2014). The plastid genome, or plastome, is considerably smaller than cyanobacterial genomes, notably due to the transfer of plastid genes to the nuclear genome (Martin et al. 2002; Bock and Timmis 2008; Bock 2017). Consequently, genes coding for subunits of the photosynthetic apparatus are segregated into both the plastid and the nuclear genomes; among the 14 PSI core subunits found in land plants, 5 (PsaA-C, PsaI, and PsaJ) are encoded in the plastid genome, and 9 are encoded in the nuclear genome (Table 1).

**Table 1.** Protein and ligand composition of PSI-LHCI in land plants^a^

Subunit	Locus	Total/Apo. (kDa)	Ligands	PSI core accumulation/photoautotrophic development in subunit-less mutants
**PSI core plastid-encoded**:				
PsaA	AtCg00350	128.7/83.2	44 Chl a (including P_700_ Chl a', A and A_0_), A_1_, F_X_, 6 β-car, 1 MGDG, 2 PG, 1 Ca^2+^	No/No (Goldschmidt-Clermont et al. 1990)
PsaB	AtCg00340	128.1/82.5	42 Chl a (including P_700_ Chl a, A and A_0_), A_1_, 5 β-car, 3 MGDG, 1 DGDG, 2 PG, 1 Ca^2+^	No/No (Girard-Bascoul et al. 1987)
PsaC	AtCg01060	9.7/9.0	F_A_, F_B_	No/No (Takahashi et al. 1991)
PsaI	AtCg00510	5.2/4.1	2 β-car	∼WT/∼WT (Schöttler et al. 2017)
PsaJ	AtCg00630	8.0/5.0	1 Chl a, 1 β-car, 1 lutein, 1 DGDG	∼75% PSI/∼WT (Schöttler et al. 2007)
**PSI core nuclear-encoded:**				
PSAD-1	At4g02770	17.9/17.9	—	No/No (Ihnatowicz et al. 2004)
PSAD-2	At1g03130	17.7/17.7		
PSAE-1	At4g28750	10.4/10.4	—	∼16% PSI, ∼130% PsaA,B/delayed (Ihnatowicz et al. 2007)
PSAE-2	At2g20260	10.5/10.5		
PSAF	At1g31330	25.0/17.3	2 Chl a, 2 β-car, 5 MGDG, 1 DGDG	∼100% PSAH, ∼60% PsaA,B, ∼10% to 20% PsaC,D,E,N/delayed (Haldrup et al. 2000; Zhang et al. 2024)
PSAG	At1g55670	17.5/11.0	3 Chl a, 1 β-car, 3 MGDG, 1 DGDG	∼80% PSI/mild delay (Varotto et al. 2002)
PSAH-1	At3g16140	12.2/10.8	1 Chl a, 1 β-car	∼WT, ∼50% PSAL/∼WT (Naver et al. 1999; Lunde et al. 2000)
PSAH-2	At1g52230	11.8/10.4		
PSAK	At1g30380	13.1/8.5	4 Chl a, 2 β-car	∼WT/∼WT (Varotto et al. 2002)
PSAL	At4g12800	21.7/17.9	3 Chl a, 2 β-car	∼WT, ∼10% PSAH/∼WT (Lunde et al. 2000)
PSAN	At5g64040	12.4/10.6	2 Chl a	∼117% PSI (but lower activity)/∼WT (Haldrup et al. 1999)
PSAO	At1g08380	11.9/10.1	2 Chl a	∼WT/∼WT (Jensen et al. 2004)
**LHCI:**				
LHCA1	At3g54890	38.8/21.6	11 Chl a, 3 Chl b, 2 β-car, 2 lutein, 1 MGDG, 1 DGDG, 1 PG	∼WT/∼WT (Ganeteg et al. 2004)
LHCA2	At3g61470	42.1/23.2	9 Chl a, 5 Chl b, 1 β-car, 1 lutein, 5 MGDG, 1 PG, 1 Vx	∼WT/∼WT (Ganeteg et al. 2004)
LHCA3	At1g61520	42.2/25.0	11 Chl a, 3 Chl b, 2 β-car, 2 lutein, 1 MGDG, 1 DGDG, 1 PG	∼WT/∼WT (Ganeteg et al. 2004)
LHCA4	At3g47470	38.6/22.3	11 Chl a, 4 Chl b, 1 lutein, 1 DGDG, 1 PG, 1 Vx	∼WT/delayed (Ganeteg et al. 2004)

^a^For each subunit, the Arabidopsis Genome Initiative (AGI) gene locus from Arabidopsis (*Arabidopsis thaliana*) is given. Note that 2 isoforms exist for PSAD, PSAE, and PSAH, but 1 isoform is present per PSI-LHCI. The sizes of the protein products from Arabidopsis were calculated with the Protein Molecular Weight tool of the sequence manipulation suite (https://www.bioinformatics.org/sms/index.html; Stothard 2000). For nucleus-encoded proteins, sizes correspond to the mature sequences after chloroplast transit peptide cleavage (and lumenal signal peptide removal for PSAF and PSAN), as predicted by TargetP-2.0 (https://services.healthtech.dtu.dk/services/TargetP-2.0/; Almagro Armenteros et al. 2019). Both the total size of the subunit including the mature apoprotein with its ligands, and the mature apoprotein alone (Apo.) are indicated. Ligands binding to each sub-units are indicated according to the PSI-LHCI structure from pea (*Pisum sativum*; PDB ID: 6YEZ; Caspy et al. 2020). Neither PSAN nor PSAO were resolved in this model, thus, their ligands are indicated according to the PSI-LHCI-LHCII structure from maize (*Zea mays*; PDB ID: 5ZJI; Pan et al. 2018). The total size of PSI-LHCI is estimated at 584 kDa, with the PSI core and LHCI belt accounting for 422 and 162 kDa, respectively. The residual amounts of PSI and the development in standard photoautotrophic conditions of mutant plants with loss (or severe decrease) of each subunit (and of both isoforms for PSAD, E and H) are indicated. For PsaA, B, and C, phenotypes in Chlamydomonas are indicated. F_X_ is bound between PsaA and PsaB (but is listed only with PsaA here), and consequently, PsaA and PsaB sizes were estimated with half an F_X_ each.

Abbreviations: A and A_0_, chlorophyll a electron donors; A1, phylloquinone; β-car, β-carotene; Chl a and b, chlorophyll a and b; F_X_, F_A_, F_B_, iron-sulfur clusters [Fe_4_-S_4_]; MGDG and DGDG, mono- and digalactosyldiacylglycerol; P_700_ Chl a and a’, chlorophyll a and chlorophyll a isomer forming the P_700_; PG, phosphatidylglycerol; Vx: violaxanthin.

Additionally, in the seed plant model Arabidopsis, the nucleus-encoded PSI subunits PSAD, PSAE, and PSAH are present as 2 isoforms each, as a result of gene duplication events (Table 1; Blanc et al. 2003). The 2 PSAD isoforms are probably functionally redundant, because the phenotype caused by the absence of *PSAD-1* can be complemented by overexpression of *PSAD-2* (Ihnatowicz et al. 2004). PSAE isoforms were initially also proposed to share a redundant function (Ihnatowicz et al. 2007); however, it was later argued that they could be beneficial under different growth conditions (Krieger-Liszkay et al. 2020). All 4 LHCI subunits (LHCA1, LHCA2, LHCA3, and LHCA4) are encoded in the nuclear genome (Table 1). Two extra LHCI subunits, LHCA5 (At1g45474) and LHCA6 (At1g19150), are lowly expressed and are not part of the structurally characterized PSI-LHCI complex (Klimmek et al. 2006; Caspy et al. 2020; Wu et al. 2023). Nevertheless, they play an important role in the formation of the PSI-NDH-PSI megacomplex, which might facilitate the electron transfer from ferredoxin to NDH in one of the proposed CEF routes (Peng et al. 2009; Otani et al. 2018; recently reviewed by Zhang et al. 2023).

### The reaction center, a plastocyanin–ferredoxin oxidoreductase

The PSI reaction center consists of the PsaA and PsaB subunits (Fig. 2B), which are thought to originate from an ancestral reaction center, likely through a gene duplication event (Büttner et al. 1992; Nelson and Junge 2015). The PsaA–PsaB heterodimer, including the ligands bound to it, amounts to 257 kDa, which accounts for more than one-half of the mass of the total PSI core of 422 kDa (Fig. 2A and Table 1). By contrast, the other PSI core subunits are rather small, ranging from 5 to 25 kDa (Table 1). PsaA–PsaB and the stromal PsaC harbor all the cofactors participating in the light-driven plastocyanin–ferredoxin oxidoreduction, namely the primary electron donor of PSI P_700_ (heterodimer of chlorophyll a and a′), the chlorophylls a A and A_0_, phylloquinone A_1_, and the 3 [Fe_4_–S_4_] clusters F_X_, F_A_, and F_B_ (Fig. 2B; Table 1; Caspy et al. 2020).

In photosynthesis, P_700_ is photoexcited to P_700_* and subsequently oxidized to P_700_^+^ by donating the electron to the chain of acceptors via either the A- or B-branch (Fig. 2B; Brettel and Leibl 2001). Then, A_1_ transfers the electron to the linear array of [Fe_4_–S_4_] clusters. PsaC is located close to the F_X_-binding site of PsaA–PsaB and contains the 2 clusters F_A_ and F_B_ (Fig. 2B and Table 1). PSAD and PSAE stabilize the attachment of PsaC to PsaA–PsaB and also support docking of ferredoxin close to PsaC, forming the stromal ridge of PSI (Fig. 2B; Antonkine et al. 2003; Caspy et al. 2020). Finally, the [Fe_2_–S_2_] of ferredoxin accepts the electron from F_B_ at the stromal side. P_700_^+^ is then reduced again by plastocyanin, which docks to the PSAF lumenal loop at the lumenal side of the PSI reaction center (Fig. 2A), bringing it in close contact with PsaA–PsaB to reduce P_700_^+^ to P_700_ (Fig. 2B; Farah et al. 1995; Haldrup et al. 1999, 2000; Amunts and Nelson 2008; Wittenberg et al. 2017).

The absence of PsaA, PsaB, PsaC, or the 2 PSAD isoforms leads to the complete loss of PSI accumulation and, consequently, photoautotrophy (Girard-Bascoul et al. 1987; Goldschmidt-Clermont et al. 1990; Takahashi et al. 1991; Ihnatowicz et al. 2004). By contrast, plants lacking both PSAE isoforms can still accumulate low levels of functional PSI-LHCI (Ihnatowicz et al. 2007). In solubilized thylakoids, both *psaN* knockout and *psaF* knockdown mutants display a severe deficiency in plastocyanin oxidation (Haldrup et al. 1999, 2000). Very slow reduction of P_700_^+^ in solubilized thylakoids was also reported for Δ*psaJ* knockout mutants of the green alga Chlamydomonas (Fischer et al. 1999) and the angiosperm tobacco (Schöttler et al. 2007). However, in vivo, redox equilibration between reduced plastocyanin, and P_700_^+^ was indistinguishable from wild-type plants in the tobacco Δ*psaJ* mutant, in line with its wild-type-like growth (Schöttler et al. 2007). This discrepancy can possibly be explained by an interaction of PsaJ and PSAF via a lumenal loop that stabilizes the plastocyanin binding site, but this might be relevant only under adverse growth conditions and may be also disturbed upon thylakoid isolation and/or detergent treatment. Similarly, despite the severe in vitro defect in P_700_^+^ reduction seen in *psaN* null mutants, also these plants exhibited wild-type-like growth and LEF (Haldrup et al. 1999), suggesting that under standard growth conditions, PSAN is dispensable for plastocyanin oxidation in vivo. By contrast, *psaF* knockdown mutants showed a severe growth defect (Haldrup et al. 2000), and for *psaF* RNAi mutants, severe defects in in vivo redox equilibration between plastocyanin and P_700_^+^ were observed (Wittenberg et al. 2017). It remains to be assessed if defects in plastocyanin oxidation and LEF in the Δ*psaJ* and the *psaN* mutants are induced in vivo by abiotic stress conditions such as heat or cold stress, which might affect protein folding and/or the stability of protein-protein interactions in PSI.

### LHCI coupling to the PSI core

Pigments bound to the PSI core (chlorophyll a and β-carotene; Table 1) and LHCI (chlorophyll a and b, β-carotene and other carotenoids; Table 1) absorb and funnel photons from the antenna to the PSI reaction center to drive electron transport. LHCI is formed by the 2 heterodimers LHCA1-LHCA4 and LHCA2-LHCA3 (Fig. 2A; Croce et al. 2002; Wientjes and Croce 2011; Wu et al. 2023). In tobacco, the Δ*psaJ* knockout mutant accumulates slightly less PSI-LHCI (approximately 80% of wild-type levels), which does not lead to any developmental defect in standard growth conditions (Schöttler et al. 2007). However, LHCI coupling to the PSI core is affected in the absence of PsaJ, and the development of the Δ*psaJ* mutant is delayed in low-light conditions. Therefore, PsaJ is important for either stable LHCI attachment to the PSI core or energy transfer from LHCI to the PSI reaction center, in agreement with its position in the PSI-LHCI complex (Fig. 2A; Schöttler et al. 2007; Wu et al. 2023). While the lumenal loop of PSAF contributes to plastocyanin docking, the hydrophobic domain of PSAF interacts with PsaJ, thus facilitating docking of LHCI to PSI (Fig. 2A; Haldrup et al. 2000; Schöttler et al. 2007; Wu et al. 2023).

PSAG and PSAK are positioned between PsaB-LHCA1 and PsaA-LHCA3, respectively (Fig. 2A; Wu et al. 2023). In a strong *psaG* antisense mutant of Arabidopsis, which did not accumulate detectable levels of PSAG, PSI content was reduced by 40% (Jensen et al. 2002). However, for an Arabidopsis *psaG* knockout mutant (resulting from an *En* transposon insertion), a less pronounced reduction in PSI content was reported. Plant development and photosynthesis in this *psaG* knockout as well as a *psaK* single knockout and the *psaG psaK* double knockout are mostly unaffected in standard growth conditions (Varotto et al. 2002). However, the *psaK* knockout shows a mild LHCI decrease, which is further aggravated in the *psaG psaK* double mutant. These findings may suggest that PSAG and PSAK buckle the LHCI belt to the PSI core (Fig. 2A).

### LHCII docking to PSI-LHCI in vascular plants

Light harvesting imbalances between PSII-LHCII and PSI-LHCI affect the redox state of the PQ pool and induce a reversible acclimation mechanism, known as state transitions (Allen 2003). In vascular plants, higher excitation rates of PSII than PSI result in the reduction of the PQ pool, which is sensed by Cyt*b*_6_*f* and activates the Cyt*b*_6_*f*-associated thylakoid protein kinase STATE TRANSITION 7 (STN7; Bellafiore et al. 2005; Shapiguzov et al. 2016; Dumas et al. 2017). STN7 phosphorylates a mobile LHCII trimer, triggering its dissociation from PSII and migration to PSI-LHCI, thus increasing the light-harvesting antenna cross-section for PSI-LHCI. In the resulting state (referred to as state 2), LHCII associates with PSI close to PSAH, PSAL, and PSAO (Lunde et al. 2000; Jensen et al. 2004; Pan et al. 2018; Wu et al. 2023). PsaI interacts with PSAH and PSAL and was proposed to stabilize them (Fig. 2A; Jensen et al. 2007; Plöchinger et al. 2016; Wu et al. 2023). However, the tobacco Δ*psaI* knockout mutant accumulates wild-type-like levels of PSI-LHCI, state transitions are not disturbed, and PSAH and PSAL are not dissociated from the complex (Schöttler et al. 2017). Only under challenging conditions (e.g. in high light and cold), and in senescent leaves, a minor PSI decrease is observed in Δ*psaI* mutants (Schöttler et al. 2017). Interestingly, PSAH accumulation in the Arabidopsis *psaL* mutant is more reduced than PSAL accumulation in the *psaH* mutant (Lunde et al. 2000). Additionally, the state transition is slightly more affected in the *psaH* than in the *psaL* mutant (Lunde et al. 2000). These findings would be compatible with a model in which PSAL mainly functions to stabilize PSAH, while PSAH directly mediates LHCII binding (Lunde et al. 2000; Jensen et al. 2007). However, the structure of PSI-LHCI-LHCII clearly shows that LHCII is relatively distant from PsaI but interacts with stromal loops of PSAL and PSAH, in line with both being involved in LHCII binding (Pan et al. 2018; Wu et al. 2023). Additionally, LHCII interacts with PSAO, which is positioned between PsaA, PSAK, and PSAL (Pan et al. 2018; Wu et al. 2023). In agreement with this observation, Arabidopsis *psaO* mutants also have a defect in state transitions (Jensen et al. 2004). Because *psaO* mutants accumulate wild-type-like amounts of PSAH and PSAL, whereas *psaH* and *psaL* mutants suffer from a strong decrease in PSAO content, PSAH and PSAL were proposed to be necessary for PSAO binding (or stabilization) to the PSI core (Jensen et al. 2004, 2007). It was later confirmed that conformational changes of PsaA, PSAK, and PSAL are necessary to stabilize PSAO in PSI-LHCI-LHCII, while PSAO may be unstable in PSI-LHCI, potentially explaining why it is not resolved in that structure (Wu et al. 2023).

For most knockout mutants of the small PSI subunits involved in LHCII binding, except for impaired state transitions, no or only mild growth defects have been described. Despite the impaired state transitions, the *psaL* mutant did not show any difference in growth and displayed only minor changes in LEF under standard growth conditions or in artificial light regimes that preferentially excited either PSI or PSII (Lunde et al. 2003). Likewise, for the *psaO* mutant, except for a slight delay in flowering, no growth defect or reduction in steady-state electron transport could be observed (Jensen et al. 2004). Notably, Gao et al. (2022) provided clear evidence that in response to chilling stress, the translation of small non-essential plastid-encoded subunits of PSII, the Cyt*b*_6_*f*, and, to a lesser extent, PSI is strongly regulated on the translational level. For one of the small Cyt*b*_6_*f* subunits, PetL, a major previously unknown function in cold acclimation of photosynthesis could be demonstrated. Whether more pronounced functional defects and growth phenotypes can be observed for nucleus-encoded and/or plastid-encoded small subunits of PSI when mutants are challenged with adverse growth conditions, especially fluctuating light and temperature stress, remains to be investigated.

## Assembly of PSI subunits and ligands

PSI-LHCI is a large multi-component complex that cannot spontaneously form or self-assemble. Rather, the complex structure of PSI-LHCI suggests a defined sequence of PSI subunit integration, from the core to the peripheral subunits (Fig. 2A). Simultaneously, nearly 240 ligands need to be incorporated into the complex at the right time and place.

**Figure 3.**
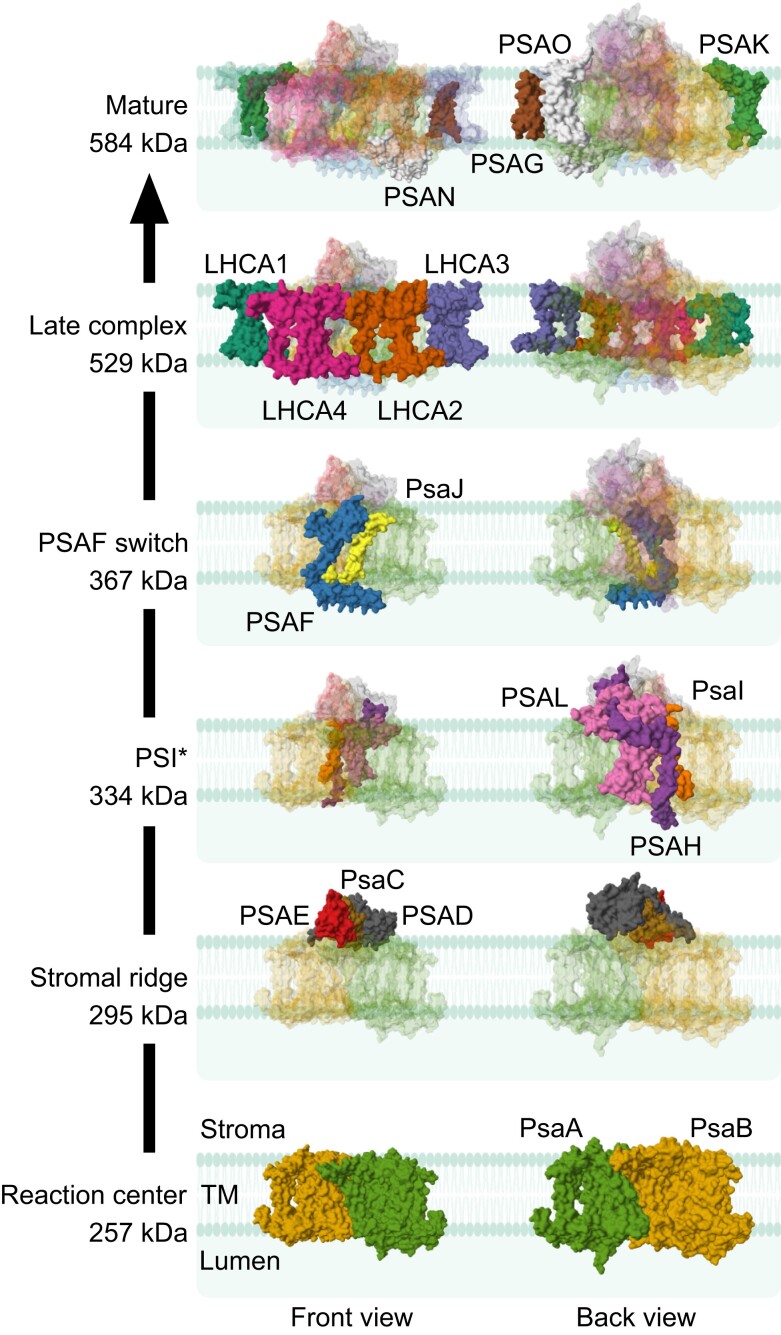
**Step-wise assembly of PSI-LHCI subunits.** First, PsaA and PsaB are co-translationally inserted into the thylakoid membrane (TM) to form the PSI reaction center. Then, PsaC, PSAD, and PSAE are incorporated to form the stromal ridge, followed by the addition of the subunits PSAH, PsaI, and PSAL, which results in the stable PSI intermediate PSI* reported in tobacco (*Nicotiana tabacum*; Wittenberg et al. 2017). Subsequently, the addition of PSAF, together with or prior to the insertion of PsaJ, triggers the maturation of PSI* by incorporation of the remaining PSI subunits and the LHCI complex, to form the PSI-LHCI supercomplex. A late PSI-LHCI intermediate was proposed to contain all PSI core subunits except PSAG and PSAK and may loosely bind LHCI in Chlamydomonas (*Chlamydomonas reinhardtii*; Ozawa et al. 2010). The sequence of incorporation of the late subunits is still largely unclear. LHCI assembly is independent of PSI core assembly. To picture the intermediate complexes, the structure of PSI-LHCI from Arabidopsis (*Arabidopsis thaliana*; PDB ID: 8J7A; Wu et al. 2023), and PSAN and PSAO from the PSI-LHCI-LHCII structure in maize (*Zea mays*; PDB ID: 5ZJI; Pan et al. 2018) were retrieved from the RCSB PDB website (https://www.rcsb.org/; Berman et al. 2000) and visualized in Mol* Viewer (https://molstar.org/viewer/; Sehnal et al. 2021). Front and back views are arbitrary. The estimated molecular weights of each subcomplex include the apoproteins with their ligands (Table 1), although ligands are not shown for clarity. The cartoon of the membrane was retrieved from BioRender (https://biorender.com/).

Below, we first briefly discuss ligand insertion into the complex and then consolidate the available information on PSI structure, phenotypes of mutants, and characterized PSI assembly factors into a step-wise assembly model of PSI (Fig. 3).

### Ligand insertion

The ligands associated with PSI are essential for light harvesting, electron transfer activity, and complex stability. They are incorporated into the subunits during protein folding and insertion into thylakoid membranes (Yang et al. 2015; Wang and Grimm 2021).

Free cofactors in the stroma are a source of ROS production (especially chlorophylls; Wang and Grimm 2015) and are prone to oxidative damage (e.g. the [Fe_4_–S_4_] clusters; Imlay 2006). Also, impaired cofactor incorporation affects protein conformation and function and induces formation of apoprotein aggregates that are degraded in the stroma or in the thylakoid membrane (Mullet et al. 1990). Synchronization of the biosynthesis and incorporation of cofactors and apoproteins is, therefore, of utmost importance to avoid the accumulation of hydrophobic apoproteins or unbound cofactors in the stroma.

Specific proteins mediate cofactor incorporation into photosynthetic complexes. Chlorophylls are highly abundant in both the PSI core and the LHCI (Table 1; Caspy et al. 2020). They bind to the nascent plastid-encoded reaction center proteins PsaA and PsaB while being co-translationally inserted into the thylakoid membranes via the membrane insertase ALBINO 3 (ALB3; Göhre et al. 2006). The nucleus-encoded LHC apoproteins imported from the cytosol are transferred by LHCP TRANSLOCATION DEFECT (LTD) to the chloroplast signal recognition particle (cpSRP) composed of the cpSRP43–cpSRP54 heterodimer (Ouyang et al. 2011; Dünschede et al. 2015; Wang and Grimm 2021). The cpSRP stabilizes LHC proteins in the stroma, thus facilitating chlorophyll incorporation. Subsequently, the cpSRP-LHC complex associates with ALB3 for integration of LHCs into the thylakoid membrane (Sundberg et al. 1997; Bellafiore et al. 2002; Wang and Grimm 2021; Rathod et al. 2022). In contrast to chlorophylls, large amounts of free carotenoids accumulate in the thylakoid membranes, where they have a major photoprotective and antioxidative function, and are believed to spontaneously incorporate into their target proteins (Dall’Osto et al. 2010; Domonkos et al. 2013; Komenda and Sobotka 2019).

Biosynthesis of the [Fe_4_–S_4_] clusters F_X_, F_A_, and F_B_ is mediated by the plastid sulfur mobilization (SUF) machinery. The proteins NITROGEN-FIXATION-SUBUNIT-U-LIKE PROTEIN 2 (NFU2; Touraine et al. 2004; Gao et al. 2013), NFU3 (Nath et al. 2017), and HIGH CHLOROPHYLL FLUORESCENCE 101 (HCF101; Lezhneva et al. 2004; Stöckel and Oelmüller 2004; Schwenkert et al. 2009) transfer F_X_ to the PsaA–PsaB heterodimer, and F_A_ and F_B_ to PsaC (Przybyla-Toscano et al. 2018; Roland et al. 2020). Incorporation of F_A_ and F_B_ into PsaC occurs before PsaC integration into the PsaA–PsaB heterodimer (Antonkine et al. 2003; Jagannathan et al. 2010).

In the absence of phylloquinone, which is a specific cofactor for PSI, its direct biosynthetic precursor 2-phytyl-1,4-naphthoquinone (Lohmann et al. 2006) or PQ (Johnson et al. 2000; Gross et al. 2006) can replace phylloquinone in the PSI complex. However, PSI-LHCI content is moderately (substitution with 2-phytyl-1,4-naphthoquinone) or strongly (substitution with PQ) reduced. In the absence of phylloquinone and very low levels of PQ, plants cannot grow photoautotrophically (Shimada et al. 2005). A model for phylloquinone biosynthesis has been derived from research in cyanobacteria, but how phylloquinone is incorporated into PSI-LHCI remains unknown (Johnson et al. 2003; Yang et al. 2015).

### Step-wise incorporation of PSI-LHCI subunits

#### From the PSI reaction center to the stable intermediate PSI*

The PSI reaction center, formed by the PsaA–PsaB heterodimer, is at the heart of PSI-LHCI (Wu et al. 2023). PsaA and PsaB are the first 2 PSI subunits to integrate into thylakoid membranes (Fig. 3). They are essential in that the lack of either PsaA or PsaB abolishes the accumulation of PSI and all of its subunits in thylakoid membranes (Girard-Bascoul et al. 1987; Goldschmidt-Clermont et al. 1990). Both proteins are co-translationally inserted into the thylakoid membranes with the help of cpSRP54 and ALB3 (Göhre et al. 2006; Hristou et al. 2019). In Chlamydomonas, PsaB was speculated to be first inserted into thylakoid membranes for subsequent anchoring of PsaA, as mutants defective in *psaB* expression do not translate *psaA*, while mutants defective in *psaA* expression are not affected in *psaB* translation (Girard-Bascoul et al. 1987; Stampacchia et al. 1997).

Integration of the stromal ridge, composed of PsaC-PSAD-PSAE, represents the second step in PSI core assembly (Fig. 3; Schöttler et al. 2011; Yang et al. 2015). Based on a model established in cyanobacteria, PsaC is probably the first protein to incorporate at the stromal side of PsaA–PsaB, followed by PSAD, and finally PSAE. PsaC association with PsaA–PsaB is unstable, and proper orientation of PsaC and stabilization of its association to PsaA–PsaB is conferred by the integration of PSAD (Li et al. 1991; Antonkine et al. 2003; Jagannathan et al. 2010). In line with these observations, PSI accumulation is completely abolished in the absence of PsaC (as shown in Chlamydomonas) and also upon absence of the 2 PSAD isoforms in Arabidopsis (Takahashi et al. 1991; Ihnatowicz et al. 2004). By contrast, low levels of PSI-LHCI, including PsaC and PSAD, accumulate in the absence of the 2 PSAE isoforms in Arabidopsis (Ihnatowicz et al. 2007), suggesting that PSAE is not absolutely essential for the assembly of the PSI reaction center. Interestingly, free PSAD and PSAE can spontaneously replace their incorporated counterparts in fully assembled PSI-LHCI in vitro (Minai et al. 2001; Lushy et al. 2002).

Subsequently, PSAH, PsaI, and PSAL are inserted into thylakoid membranes at the periphery of the PSI reaction center (Figs. 2A and 3). Together, the PsaA–PsaB, PsaC-PSAD-PSAE, and PSAH-PsaI-PSAL modules form a relatively stable PSI subcomplex that has been characterized in tobacco and named PSI* (Fig. 3; Wittenberg et al. 2017). The faster electrophoretic migration of PSI* compared with PSI-LHCI, as observed in blue-native polyacrylamide gel electrophoresis (BN-PAGE), had previously been attributed to the absence of LHCI from PSI*, and therefore, PSI* had been annotated as PSI core (Järvi et al. 2011). However, PSI* subunit characterization by immunoblotting and mass spectrometry revealed that several core subunits are absent from this complex, most notably PSAF (Wittenberg et al. 2017). Despite the interaction of PSAO with the PSAH-PsaI-PSAL module, PSAO was not detected in PSI* (Wittenberg et al. 2017). It is currently unclear whether PSAO is incorporated at this stage or later in the assembly process, although it should be noted that not only PsaA and PSAL, but also PSAK, which is not yet present at this step, are important for stable association of PSAO with the PSI core (Wu et al. 2023). The ratio of PSI* to mature PSI-LHCI is higher in young leaf tissue compared with older tissues, in line with PSI* being a PSI assembly intermediate accumulating mostly during chloroplast biogenesis (Wittenberg et al. 2017).

#### From PSI* to the mature PSI-LHCI

Steps involved in the maturation of PSI* to mature PSI-LHCI are not critically dependent on the presence of PSAH, PsaI, and PSAL (Lunde et al. 2000; Schöttler et al. 2017). By contrast, PSAF is essential for PSI-LHCI accumulation in Arabidopsis (Haldrup et al. 2000), and the PSI* to PSI-LHCI ratio in inducible *PSAF* RNA interference (RNAi) lines in tobacco increases upon induction of the *PSAF* knockdown (Wittenberg et al. 2017). PSI* contains all redox-active cofactors and exhibits electron transport activity similar to mature PSI-LHCI. However, analysis of the *PSAF* RNAi tobacco line, which overaccumulates PSI*, revealed a less efficient plastocyanin oxidation by PSI* than by PSI-LHCI (Wittenberg et al. 2017). This is consistent with the absence of PSAF from PSI*, whose lumenal loop mediates plastocyanin docking (Haldrup et al. 2000; Caspy et al. 2020). Therefore, incorporation of PSAF was considered to represent the rate-limiting step of PSI maturation at the stage of the PSI* intermediate (Fig. 3; Wittenberg et al. 2017).

A PSI assembly intermediate containing the PSI* subunits and PSAF was recently proposed to originate from PSI* (or a PSI*-like complex) in Arabidopsis (Zhang et al. 2024). The subsequent steps in the maturation of PSI*-PSAF to PSI-LHCI are largely unknown. Based on the structural data for PSI-LHCI, and the finding that the Δ*psaJ* mutant in tobacco is affected in LHCI coupling to the PSI core, PsaJ is likely inserted after PSAF, but before the addition of the LHCI belt (Figs. 2A and 3; Schöttler et al. 2007; Caspy et al. 2020). An additional complex intermediate between PSI* and the mature PSI-LHCI was characterized in Chlamydomonas. It contains all PSI* subunits and weakly binds PSAF and PsaJ (Ozawa et al. 2010). In view of the absence of LHCI from the purified complex, it was suggested that additional subunits may be necessary to stabilize the LHCI to an extent that allows its retention upon detergent treatment and during complex isolation (Ozawa et al. 2010).

LHCI is formed by the 2 heterodimers LHCA1-LHCA4 and LHCA2-LHCA3 (Croce et al. 2002; Wientjes and Croce 2011; Wu et al. 2023). It is currently unclear whether they assemble as full LHCI complex, as 2 separate heterodimers in the stroma, or during LHCA protein insertion into the thylakoid membrane, before association with the PSI core (Fig. 3; Wittenberg et al. 2017; Rathod et al. 2022). Nonetheless, LHCI is thought to be assembled independently of the PSI core. Reinforcing this hypothesis, mutants suffering from complete loss or strong reduction of the PSI core are only mildly affected in LHCI subunit accumulation (e.g. the *hcf101* (Stöckel and Oelmüller 2004), *ppd1* (Liu et al. 2012), and Δ*ycf4* (Krech et al. 2012) mutants). Mutants suffering from partial loss of PSI have a typical phenotype in chlorophyll-*a* fluorescence at low temperature (77 K), with the PSI-LHCI maximum emission signal (733 nm) being blue-shifted due to the presence of LHCI that is not coupled to the PSI core, resulting in a stronger signal in the 705 to 720 nm range due to increased emission from free LHCI (e.g. Stöckel and Oelmüller 2004; Stöckel et al. 2006; Schöttler et al. 2007; Krech et al. 2012).

The addition of the PSAG and PSAK subunits likely follows during the assembly of PSI-LHCI (Fig. 3). PSAG and PSAK can spontaneously insert into fully assembled PSI-LHCI in vitro (Mant et al. 2001; Zygadlo et al. 2006), although the situation might be different in vivo (Fristedt et al. 2014; Wittenberg et al. 2017). Although PSAG and PSAK are absent from the late intermediate in Chlamydomonas (Ozawa et al. 2010), the possibility of an LHCI-PSAG subcomplex forming before LHCI association to the PSI core was suggested in tobacco (Wittenberg et al. 2017). The position of PSAG and PSAK on opposite sides of LHCI and the more severe LHCI decrease when both proteins are absent from Arabidopsis suggest that these 2 subunits might stabilize LHCI during or after its association to the PSI core (Figs. 2A and 3; Varotto et al. 2002; Ozawa et al. 2010; Wu et al. 2023).

PSAN accumulates normally in the absence of both PSAG and PSAK (Varotto et al. 2002); in the absence of PSAN, plant development is similar to the wild type, and normal amounts of LHCI accumulate. Thus, PSAN incorporation appears to occur independently of PSAG and PSAK, and PSAN could be the last subunit to integrate upon formation of the mature PSI-LHCI complex (Fig. 3).

## The machinery for PSI-LHCI subunit assembly

**Figure 4.**
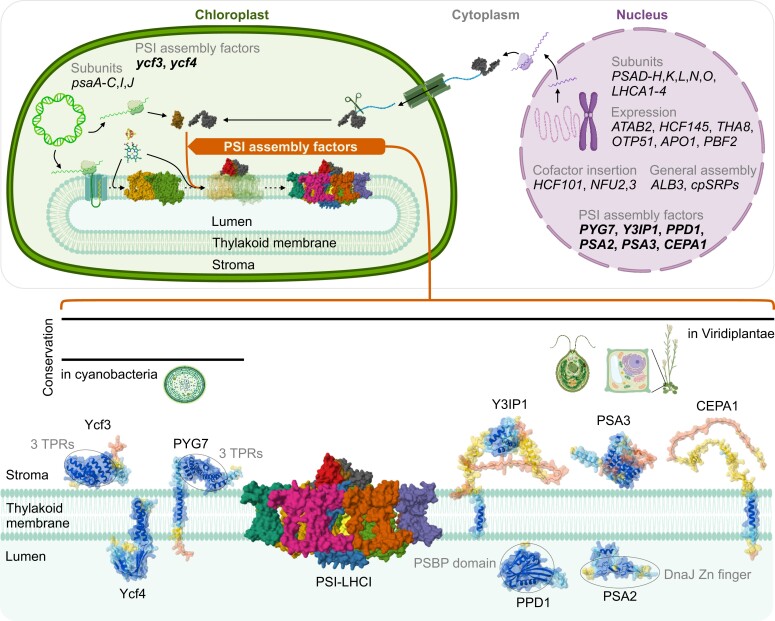
**PSI-LHCI assembly is controlled at multiple levels.** PSI-LHCI assembly takes place in thylakoid membranes in the chloroplast. The plastid genome encodes a set of PSI subunits and assembly factors (AFs; *ycf3* and *ycf4*), while nucleus-encoded genes code for the rest of the PSI subunits, all LHCI subunits, and the majority of PSI assembly factors (*PYG7*, *Y3IP1*, *PPD1*, *PSA2*, *PSA3*, and *CEPA1*), but also numerous proteins involved in the regulation of the expression of PSI-related plastid genes, PSI cofactor biosynthesis as well as accessory proteins involved in membrane insertion of subunits and/or complex assembly that are shared by both photosystems. Nucleus-encoded proteins are translated in the cytosol and possess an N-terminal chloroplast transit peptide (cTP), targeting them to the chloroplast via the translocon complexes in the chloroplast envelope. Post-import cleavage of the cTP (indicated by scissors) gives rise to mature protein. PSI assembly initiates with co-translational insertion of the plastid-encoded PsaA and PsaB into the thylakoid membrane. PSI cofactor (e.g. [Fe_4_-S_4_] clusters and chlorophylls) and subunit insertion are highly coordinated to yield the mature PSI-LHCI. PSI subunit incorporation is mediated by the PSI assembly machinery, composed of PSI assembly factors. Here, the PSI assembly factors described for land plants are shown. Ycf3, Ycf4, and PYG7 are conserved from cyanobacteria to Viridiplantae, while other PSI assembly factors appeared later in evolution and are specific to the eukaryotic lineage (Table 2). The soluble Ycf3 and PSA3 in the stroma and PPD1 and PSA2 in the lumen are anchored to the thylakoid membrane during PSI assembly. Other PSI assembly factors are embedded in the thylakoid membrane *via 2* (Ycf4) or 1 (PYG7, Y3IP1, and CEPA1) transmembrane domains. While Ycf4 topology has not yet been confirmed experimentally, the C terminus of PYG7 and the N terminus of Y3IP1 and CEPA1 are known to represent stromal soluble regions. Ycf3 and PYG7 harbor 3 tetratricopeptide repeat (TPR) domains, PPD1 harbors a PSBP domain, and PSA2 harbors a DnaJ-like zinc (Zn) finger domain. The mature PSI-LHCI and the PSI assembly factors are drawn to scale. The structure of PSI-LHCI in Arabidopsis (PDB ID: 8J7A; Wu et al. 2023) was retrieved from the RCSB PDB website (https://www.rcsb.org/; Berman et al. 2000). AlphaFold structural predictions of Arabidopsis Ycf3 (AF-P61843-F1), Ycf4 (AF-P56788-F1), PYG7 (AF-B9DHG0-F1), Y3IP1 (AF-Q9LU01-F1), PPD1 (AF-O23403-F1), PSA2 (AF-O64750-F1), PSA3 (AF-Q9M3C6-F1) and CEPA1 (AF-Q9LY44-F1) were retrieved from the AlphaFold Protein Structure Database (AlphaFold DB; https://alphafold.ebi.ac.UK/; Jumper et al. 2021; Varadi et al. 2022). All structures were visualized in Mol* Viewer (https://molstar.org/viewer/; Sehnal et al. 2021). cTP sequences (and signal peptide sequences for lumenal localization of PYG7, PPD1 and PSA2) were predicted by TargetP-2.0 (https://services.healthtech.dtu.dk/services/TargetP-2.0/; Almagro Armenteros et al. 2019) and removed from the predicted structures of the PSI assembly factors. Cartoons of the nucleus, chromosome, plastid genome, ribosomes and transcripts, translocons, membrane, cyanobacterium, Chlamydomonas, Arabidopsis, and the plant cell were retrieved from BioRender (https://biorender.com/).

Assembly of the PSI-LHCI complex needs to be tightly coordinated in time and space (Schöttler et al. 2011; Yang et al. 2015). Moreover, maintenance of the proper stoichiometry between proteins and ligands in the stroma, but also between plastid-encoded and nucleus-encoded proteins, is crucial. Folding and incorporation of the different subunits needs to follow the step-wise assembly pathway compatible with the PSI-LHCI structure, as described above (Fig. 3). Additionally, PSI assembly intermediates need to be stabilized and protected, especially from photo-oxidative damage at sensitive sites such as cofactors and reduced residues, but also from proteolytic attack to exposed subunit domains. For all these reasons, PSI-LHCI assembly is critically dependent on a sophisticated machinery that acts at multiple levels to safeguard undisturbed PSI biogenesis (Fig. 4). Subunit synthesis is regulated at the transcriptional and/or translational levels in the nucleus and the plastid to control protein stoichiometry and is additionally controlled at the post-translational level (e.g. by protein turnover).

### Regulation of PSI-related protein synthesis

PSI-LHCI protein content is dependent on both plastid and nuclear gene expression and their regulation at the transcriptional and post-transcriptional levels. In land plants, in addition to the 5 PSI subunits PsaA, PsaB, PsaC, PsaI, and PsaJ, 2 proteins mediating PSI-LHCI assembly are encoded in the plastid genome: Ycf3 and Ycf4 (discussed below; Shinozaki et al. 1986; Ruf et al. 1997; Krech et al. 2012). An exception was found in the genus *Lathyrus*, a legume, which lost both *psaI* and *ycf4* from the plastid genome, without any identifiable copy in the nuclear genome (Magee et al. 2010). Plastid gene expression is mainly regulated at the post-transcriptional level, as opposed to cyanobacteria (Zoschke and Bock 2018). An autoregulation mechanism for plastid-encoded photosynthesis-related genes, known as control by epistasy of synthesis (CES), was reported in Chlamydomonas (Choquet and Vallon 2000). CES regulates the translation of subunits in the absence of other essential subunits of the same protein complex, thus reducing the accumulation of free proteins in the stroma. In the case of PSI, the absence of PsaB leads to downregulation of *psaA* expression, and the absence of PsaA leads to downregulation of *psaC* expression (Wostrikoff et al. 2004). In addition, several proteins promoting the translation of PSI-related plastid transcripts were identified. This set of proteins includes the Arabidopsis TAB2 (ATAB2), initially proposed to be an activator of PSI and PSII reaction center subunit translation, although its mode of action may need to be revised (Barneche et al. 2006; Belcher et al. 2015), and HCF145, involved in transcript stabilization of the *psaA*-*psaB*-*rps14* transcription unit (Lezhneva and Meurer 2004; Manavski et al. 2015). Ycf3 is encoded by a gene containing 2 group II introns, and, therefore, *ycf3* transcript maturation is dependent on intron splicing factors such as THYLAKOID ASSEMBLY 8 (THA8; Khrouchtchova et al. 2012), ORGANELLE TRANSCRIPT PROCESSING 51 (OTP51; De Longevialle et al. 2008), and ACCUMULATION OF PHOTOSYSTEM ONE 1 (APO1; Watkins et al. 2011). Moreover, PHOTOSYSTEM I BIOGENESIS FACTOR 2 (PBF2) was shown to be specifically involved in *ycf3* intron 1 splicing (Wang et al. 2020). In addition to the stoichiometry of the plastid-encoded subunits, the ratio between plastid-encoded and nucleus-encoded subunits is also of critical importance. Therefore, plastid-to-nucleus retrograde signaling is essential to regulate the expression of photosynthesis-associated nuclear genes whose products are involved in chloroplast biogenesis as well as the import of these gene products into the chloroplast (Hernández-Verdeja and Strand 2018; Wu et al. 2018, 2019; Wu and Bock 2021).

### PSI assembly factors

The machinery for photosynthetic complex assembly is composed of auxiliary proteins, referred to as assembly factors, which are not stably associated with the mature complexes but instead mediate subunit incorporation into the thylakoid membrane and/or proper association of the different subunits with each other. Because all photosynthetic complexes share similarities (in that they, e.g., are complexes formed of proteins and cofactors, reside in thylakoid membranes, and are interdependent in photosynthetic electron transfer), their assembly machineries can partially overlap in that some assembly factors participate in the biogenesis of more than 1 complex (e.g. ALB3 Bellafiore et al. 2002; Göhre et al. 2006, and ALB4 Benz et al. 2009; Trösch et al. 2015). However, because the abundances of the different thylakoidal protein complexes are regulated differently (Schöttler and Tóth 2014), and they are composed of different sets of subunits, there are specific sets of assembly factors for each complex. For instance, DECREASED ELECTRON TRANSPORT AT PSII (DEAP2; Keller et al. 2023), DE-ETIOLATION INDUCED PROTEIN 1 (DEIP1; Sandoval-Ibáñez et al. 2022), and CONSERVED ONLY IN THE GREEN LINEAGE 160 (CGL160; Rühle et al. 2014; Fristedt et al. 2015; Reiter et al. 2023) are the latest factors shown to be specifically involved in the assembly of PSII, Cyt*b*_6_*f*, and the chloroplast ATPase, respectively.

The machinery that specifically mediates PSI biogenesis comprises numerous PSI assembly factors (Fig. 4 and Table 2), and it appears likely that there are additional factors that remain to be discovered. PSI assembly factors are characterized by 6 main features: (1) they are plastid-localized proteins that are enriched in the stroma lamellae together with PSI-LHCI, but they are not necessarily intrinsic membrane proteins; (2) they are not an integral and stable part of the PSI-LHCI complex; (3) they are specifically involved in the accumulation of PSI subunits; (4) they do not directly regulate plastid gene expression; (5) instead, they mediate the folding, incorporation, stabilization, and/or protection of PSI subunits during complex biogenesis; and (6) they are usually coexpressed with PSI biogenesis, especially in young greening leaf tissues. To date, 8 proteins have been characterized as PSI assembly factors in land plants: Ycf3 (Ruf et al. 1997), Ycf4 (Krech et al. 2012), PYG7 (Stöckel et al. 2006; Yang et al. 2017), Y3IP1 (Albus et al. 2010), PPD1 (Liu et al. 2012; Roose et al. 2014), PSA2 (Fristedt et al. 2014; Wang et al. 2016), PSA3 (Shen et al. 2017), and CEPA1 (Rolo et al. 2024; also named PBF8; Zhang et al. 2024; Fig. 4 and Table 2). All of them are conserved in green algae, but only Ycf3, Ycf4, and PYG7 are present also in cyanobacteria (Fig. 4 and Table 2). By contrast, a recently described PSI assembly factor, Ycf51, is found (nearly) exclusively in cyanobacteria (Table 2; Dai et al. 2024). The role of these proteins in PSI assembly is discussed below.

**Table 2.** PSI assembly factors^a^

Arabidopsis						Chlamydomonas			Synechocystis	
Name	Locus	UniProt	CS	TM	Size (kDa)	Name	Locus	GreenCut2	Name	Locus
Ycf3 Hypothetical chloroplast open reading frames number 3	AtCg00360	P61843	No	No	19.5	Ycf3/PafI PSI assembly factor I	CreCp.g802301	No	Ycf3	slr0823
Ycf4 Hypothetical chloroplast open reading frames number 4	AtCg00520	P56788	No	F22-T40; G63-L85	21.4	Ycf4/PafII	CreCp.g802302	No	Ycf4	sll0226
PYG7 PALE YELLOW GREEN 7	At1g22700	B9DHG0	A101-A102	I121-I141	22.7	CGL71 CONSERVED ONLY IN THE GREEN LINEAGE 71	Cre12.g524300	Yes	Ycf37	slr0171
Y3IP1 Ycf3-INTERACTING PROTEIN 1	At5g44650	Q9LU01	C37-K38	A255-F275	27.9	Y3IP1/CGL59	Cre06.g280650	Yes	—	—
PPD1 PSBP-DOMAIN PROTEIN 1	At4g15510	O23403	A104-S105	No	21.3	PSBP4 PSBP-LIKE PROTEIN 4	Cre08.g362900	Yes	—	—
PSA2 PHOTOSYSTEM I ASSEMBLY 2	At2g34860	O64750	A93-L94	No	10.1	ZNJ1 DnaJ-LIKE ZINC-FINGER PROTEIN 1	Cre11.g475850	Yes	—	—
PSA3 PHOTOSYSTEM I ASSEMBLY 3	At3g55250	Q9M3C6	A46-Y47	No	26.2	PSA3	Cre03.g183400	No	—	—
CEPA1 CO-EXPRESSED WITH PSI ASSEMBLY 1 /PBF8 PHOTOSYSTEM I BIOGENESIS FACTOR 8	At3g56010	Q9LY44	C51-L52	I170-W192	16.3	PIR1 PHOTOSYSTEM I REQUIRED 1 /LGS1 LIGHT GROWTH SENSITIVE 1	Cre01.g014000	No	—	—
—	—	—	—	—	—	—	—	No	Ycf51	sll1702

^a^Homologs in Arabidopsis (*Arabidopsis thaliana*; land plant model), Chlamydomonas (*Chlamydomonas reinhardtii*; green algal model), and Synechocystis (*Synechocystis* sp. PCC 6803; cyanobacterial model) are reported. The Uniprot ID is indicated for each Arabidopsis protein (https://www.uniprot.org/; The UniProt Consortium 2021). The chloroplast transit peptide cleavage site and the signal peptide cleavage site for lumenal localization of PYG7, PPD1, and PSA2 were predicted by TargetP-2.0 (https://services.healthtech.dtu.dk/services/TargetP-2.0/; Almagro Armenteros et al. 2019). Transmembrane domains of the mature sequences were predicted by DeepTMHMM (https://dtu.biolib.com/DeepTMHMM; Hallgren et al. 2022). Sizes of mature proteins were calculated with the Protein Molecular Weight tool of the sequence manipulation suite (https://www.bioinformatics.org/sms/index.html; Stothard 2000). Precursor protein sizes are 33.7 kDa for PYG7, 31.8 kDa for Y3IP1, 32.3 kDa for PPD1, 19.9 kDa for PSA2, 31.2 kDa for PSA3, and 21.9 kDa for CEPA1. Conservation in the GreenCut2 set is indicated (Karpowicz et al. 2011). The absence of a homolog is indicated by —.

Abbreviations: CS, cleavage site; TM, transmembrane domain.

#### Ycf3

The first PSI assembly factors reported in photosynthetic organisms are encoded by the *hypothetical chloroplast open reading frames number 3* and *4* (*Ycf3* and *Ycf4*; Wilde et al. 1995; Boudreau et al. 1997; Ruf et al. 1997). They are the only 2 plastid-encoded PSI assembly factors (Fig. 4 and Table 2) and are present in distinct gene clusters within the plastid genomes of land plants: *ycf3* resides downstream of the *rps14-psaA-psaB* operon encoding a plastid ribosomal subunit and the 2 reaction center proteins of PSI, and *ycf4* is part of an operon encoding the small PSI subunit PsaI, an envelope protein potentially involved in proton exchange, and the cytochrome *f* subunit (PetA) of Cyt*b*_6_*f* (*psaI-ycf4-cemA* (*ycf10*)*-petA*; Shinozaki et al. 1986; Ruf et al. 1997; Krech et al. 2012; Trinh et al. 2021).

Sequencing of the tobacco plastome (Shinozaki et al. 1986) as well as development of an efficient transformation method for the tobacco plastid genome (Svab et al. 1990; Svab and Maliga 1993) enabled the characterization of predicted open reading frames (ORFs) of unknown function. In that context, Ycf3 was not only the first described PSI assembly factor in land plants but also the first plastid ORF of unknown function to be inactivated in seed plants by a reverse genetic approach (Ruf et al. 1997).

The disruption of *ycf3* led to the complete and specific loss of PSI in tobacco, without any defects in plastid transcription, mRNA stability, or translation (Ruf et al. 1997). Similarly, Δ*ycf3* knockout mutants in Chlamydomonas and the cyanobacterium Synechocystis (*Synechocystis* sp. PCC 6803) do not accumulate PSI, and cells are unable to grow photoautotrophically (Boudreau et al. 1997; Schwabe 2003; Schwabe et al. 2003). A 50% reduction of Ycf3 accumulation due to inefficient removal of the second intron in a deletion mutant of the first *ycf3* intron resulted in a 50% reduction in PSI content when plants were grown in high light (Petersen et al. 2011). Likewise, a barley (*Hordeum vulgare*) mutant suffering from a temperature-dependent defect in intron 1 splicing and maturation of the *ycf3* mRNA suffered from a strong decrease in Ycf3 protein and PSI accumulation at elevated temperature (Landau et al. 2009). Taken together, these data suggest that Ycf3 abundance may be a limiting factor in PSI assembly.

Ycf3 contains 3 tetratricopeptide repeat (TPR) motifs in its sequence, which are known to be involved in mediating protein-protein interactions (Fig. 4; Lamb et al. 1995; Bohne et al. 2016). In Chlamydomonas, co-immunoprecipitation (co-IP) assays suggested interaction of Ycf3 with PsaA and PSAD, probably via the TPR motifs, as point mutations in these domains led to impaired growth and photosynthetic activity (Naver et al. 2001). Ycf3 is a soluble stromal protein (Fig. 4 and Table 2) that interacts with newly synthesized PsaA and thus is essential for early PsaA-PsaB heterodimer assembly (Nellaepalli et al. 2018, 2021).

#### Ycf4

The first Δ*ycf4* knockout was reported in Synechocystis, where inactivation of *ycf4*/*orf184* decreased the PSI content to approximately 80% of wild-type levels and increased the PSII content 2 to 3 times compared with that of the wild type (Table 2; Wilde et al. 1995). Despite the imbalance in the PSI to PSII ratio, Δ*ycf4* knockout cells grow similar to the wild type (Wilde et al. 1995). In tobacco, the Δ*ycf4* knockout mutant accumulates only approximately 10% of wild-type PSI levels, allowing photoautotrophic growth despite a severely delayed plant development (Krech et al. 2012). Therefore, as opposed to Ycf3, Ycf4 is non-essential for PSI accumulation in Synechocystis and tobacco, even though loss of Ycf4 seems to be more deleterious in the latter organism (Wilde et al. 1995; Ruf et al. 1997; Schwabe 2003; Schwabe et al. 2003; Krech et al. 2012). By contrast, the phenotype of the Δ*ycf4* knockout in Chlamydomonas is more severe than in cyanobacteria and land plants, as cells cannot grow photoautotrophically (Boudreau et al. 1997). Additionally, Chlamydomonas cells expressing a mutated version of Ycf4 (E179/181Q) that harbors 2 amino acid substitutions in the soluble protrusion of the protein (Fig. 4) exhibit an unstable PSI reaction center due to a downstream block in PSI assembly (Onishi and Takahashi 2009). Co-IP followed by mass spectrometry and immunoblot assays in Chlamydomonas revealed that Ycf4 interacts with a large complex containing at least the PSI reaction center PsaA–PsaB, the stromal ridge PsaC-PSAD-PSAE, and PSAF (Ozawa et al. 2009). Additionally, Ycf4 oligomers (proposed to form the so-called Ycf4 module) associate with newly synthesized PSI reaction centers but also with LHCI subunits (Nellaepalli et al. 2018). In tobacco, Ycf4 co-migrates with a complex slightly below mature PSI-LHCI in native gel electrophoresis (Krech et al. 2012), supporting a role of Ycf4 ranging from the early steps of PSI assembly potentially all the way to LHCI incorporation. Consistent with its relatively high stability as revealed by pulse-chase labeling experiments, Ycf4 is probably reused rather than quickly degraded after having fulfilled its function (Nellaepalli et al. 2018). The Ycf4 module has been proposed to serve as a scaffold to facilitate subunit integration during PSI assembly and protect PSI subcomplexes (Nellaepalli et al. 2018, 2021).

Recently, the generation of another Δ*ycf4* knockout mutant in tobacco (here referred to as Δ*ycf4-full* to distinguish it from the Δ*ycf4* mutant reported in Krech et al. 2012) was claimed to be unable to grow on soil due to loss of photoautotrophy, thus contradicting previous observation (Krech et al. 2012; Khan et al. 2022). The different phenotypes were attempted to be explained by the fact that, in the original Δ*ycf4* knockout mutant generated by Krech et al. (2012), only the first 93 amino acids of Ycf4 (M1-D93) were removed and replaced by *aadA* selectable marker gene cassette (encoding the enzyme aminoglycoside 3″-adenylyltransferase that confers spectinomycin resistance), while in the Δ*ycf4-full* mutant, the whole *ycf4* gene was replaced by a cassette containing both *aadA* and *GREEN FLUORESCENT PROTEIN* (*GFP*) sequences. However, it seems clear that the original Δ*ycf4* mutant represents a full gene knockout, as Ycf4 was shown to be absent from Δ*ycf4* by immunoblotting using polyclonal antibodies raised against 2 peptides, one of which (T157-A170) was derived from the nondeleted region (Krech et al. 2012). These antibodies should allow detection of a putative N-terminally truncated Ycf4Δ1-93 protein, which, moreover, would lack the 2 transmembrane domains essential for thylakoid membrane integration. Instead, the reported phenotype of the Δ*ycf4-full* phenotype may be the result of improper growth conditions, the orientation of the transgene cassette and/or the additional metabolic burden resulting from high-level GFP accumulation, which may aggravate the already severe phenotype resulting from the loss of Ycf4 function. Thus, caution needs to be exercised before claiming novel functions for Ycf4 that are merely based on predicted Ycf4 protein interactions but have not been confirmed experimentally (Khan et al. 2022). However, based on findings in Chlamydomonas where an eyespot protein was identified as putative interactor of Ycf4 (Ozawa et al. 2009), it currently cannot be excluded that Ycf4 exerts an additional function related to the eyespot (which is absent from land plant chloroplasts).

#### PYG7 (CGL71, Ycf37)

As TPR motif-containing proteins often mediate the assembly of protein complexes (Ruf et al. 1997; Bohne et al. 2016). Wilde et al. (2001) conducted a bioinformatic screen for TPR-containing proteins in the cyanobacterium Synechocystis. This led to the identification of Ycf37, which was subsequently inactivated by reverse genetics. Growth of Δ*ycf37* knockout mutants was largely uncompromised despite a decreased PSI to PSII ratio. As opposed to the Synechocystis Δ*ycf4* knockout, which exhibits a similar decrease in PSI amounts, the PSII content was not increased in the Δ*ycf37* mutant strain (Wilde et al. 1995, 2001). Therefore, Ycf37 was proposed to be a non-essential factor mediating PSI assembly and/or stability (Wilde et al. 2001). Because the Δ*ycf37* mutant accumulates PSI monomers, including a PSI subcomplex, it was later suggested that Ycf37 might protect the PSI subcomplex and mediate PSI trimerization in cyanobacteria (Dühring et al. 2006).

The *ycf37* gene is not conserved in the plastomes of photosynthetic eukaryotes. Instead, orthologs in Arabidopsis and Chlamydomonas known as *PALE YELLOW GREEN 7* (*PYG7*; Fig. 4; Stöckel et al. 2006) and *CONSERVED ONLY IN THE GREEN LINEAGE 71* (*CGL71*; Heinnickel et al. 2016), respectively, are present in the nuclear genomes of plants and algae and code for proteins that are imported into the chloroplast via their N-terminal chloroplast transit peptide (cTP) sequence (Table 2). In Arabidopsis, a *pyg7-1* knockout mutant was isolated by ethyl methanesulfonate–mediated mutagenesis. The *pyg7-1* mutant is unable to grow photoautotrophically and develops pale yellow-green cotyledons (Stöckel et al. 2006). When grown heterotrophically on synthetic medium, *pyg7-1* development is strongly delayed, and the seedlings exhibit small pale yellow-green leaves, resulting from the loss of PSI core subunit accumulation. Therefore, in contrast to Ycf37 in cyanobacteria, PYG7 is essential for PSI accumulation in land plants.

The Chlamydomonas ortholog CGL71 belongs to the GreenCut2 list (Merchant et al. 2007; Karpowicz et al. 2011), which comprises Chlamydomonas proteins conserved in photosynthetic but not in nonphotosynthetic organisms. GreenCut2 proteins are, therefore, thought to have functions related to photosynthesis. In Chlamydomonas, *cgl71* knockout mutants cannot grow on minimal medium (Heinnickel et al. 2016). When grown in the presence of a reduced carbon source (i.e. on Tris-acetate-phosphate medium) and at low-light intensity (30 *µ*mol photons m^−2^ s^−1^), *cgl71* cells grew similar to the wild type but accumulated only approximately 30% of wild-type levels of PSI per chlorophyll. The decrease in PSI accumulation in *cgl71* cells was shown to be caused by oxidative stress rather than photodamage. Therefore, the CGL71 protein was proposed to protect PSI during its assembly, in line with the more severe phenotype in eukaryotes than in prokaryotes, where the photosynthetic apparatus is likely assembled in a less oxic environment, because the O_2_-consuming respiration and photosynthetic complex assembly take place in the same membrane system (Heinnickel et al. 2016). Indeed, PSI intermediates with exposed [Fe_4_–S_4_] clusters F_X_, F_A_, and F_B_ incorporated either at the stromal side of PsaA–PsaB (F_X_) or within PsaC (F_A_ and F_B_) are particularly sensitive to oxidation (Chen et al. 2022b). More recently, PYG7 was shown to interact with PsaC in Arabidopsis, presumably via its stromal TPR-containing region (Fig. 4; Yang et al. 2017). Although originally predicted to integrate into the thylakoid membrane with 2 consecutive short transmembrane domains (Yang et al. 2017), these proposed domains likely represent a single transmembrane span based on the size of the hydrophobic stretch and DeepTMHMM and AlphaFold predictions (Fig. 4; Table 2). Although PsaC was not co-immunopurified with CGL71-containing complexes in Chlamydomonas, CGL71 was proposed to protect the [Fe_4_–S_4_] cluster at the stromal side of PsaA–PsaB until integration of the PsaC-PSAD-PSAE stromal ridge occurs (Nellaepalli et al. 2021).

#### Y3IP1

Ycf3 was shown to interact with PsaA and PSAD in Chlamydomonas (Naver et al. 2001), but for a long time, no Ycf3-interacting proteins could be identified in tobacco. Because no antibodies could be successfully raised against Ycf3 in tobacco, Ycf3-FLAG expressing tobacco lines, which complemented the Δycf3 knockout, were generated by stable chloroplast transformation (Bock 2015) and used for the isolation of Ycf3-interacting proteins by co-IP coupled to mass spectrometry (Albus et al. 2010). Ycf3-INTERACTING PROTEIN 1 (Y3IP1) was co-purified with Ycf3-FLAG and validated as a new PSI assembly factor in tobacco and Arabidopsis (Fig. 4 and Table 2; Albus et al. 2010). In both organisms, *y3ip1* knockdown mutants can still accumulate low amounts of PSI. Although no *y3ip1* null mutant was available, the most severe tobacco RNAi lines were unable to grow photoautotrophically, suggesting that Y3IP1 may be essential for PSI assembly (Albus et al. 2010). However, Y3IP1 is not essential for Ycf3 accumulation and its anchoring to thylakoid membranes (Albus et al. 2010). Y3IP1 is conserved in green algae and land plants but, unlike Ycf3, absent from cyanobacteria (Fig. 4; Table 2; Boudreau et al. 1997; Ruf et al. 1997; Schwabe 2003; Schwabe et al. 2003). Consequently, Y3IP1 likely co-evolved with PSI assembly in photosynthetic eukaryotes and might be connected to PSI assembly mechanisms that differ from those in prokaryotes. Given their direct physical interaction, Ycf3 and Y3IP1 were proposed to cooperate in PSI assembly (Albus et al. 2010). Indeed, it was later shown in Chlamydomonas that both proteins form the so-called Ycf3-Y3IP1 module, which associates with newly synthesized PsaA–PsaB (Nellaepalli et al. 2018). More precisely, Ycf3 may assist with the early assembly of PsaA–PsaB, while Y3IP1 may mediate the translocation of the PSI reaction center from the Ycf3 to the Ycf4 module (Nellaepalli et al. 2021). Notably, pulse-chase labeling assays suggested that Ycf3 turnover is rather fast, and the protein may be rapidly degraded after use, while Y3IP1 is relatively stable and, therefore, might be reused several times in a similar manner as Ycf4 (Nellaepalli et al. 2018). Interestingly, overexpression of Y3IP1 in Arabidopsis and of its homolog in rice (*Oryza sativa*), CHLOROPLAST PROTEIN-ENHANCING STRESS TOLERANCE (CEST, OsY3IP1), resulted in increased plant tolerance to multiple abiotic stresses, including salt stress, drought stress, heat stress, and oxidative stress induced by methylviologen, which catalyzes ROS production at the PSI acceptor side (Yokotani et al. 2011; Moon et al. 2022). However, it remains to be determined if this is attributable to altered PSI accumulation before the stress treatments or to a different capacity of the plants to recover from damage to PSI.

The recent identification of a Ycf3-interacting protein in cyanobacteria (Ycf51; Dai et al. 2024; Table 2) has prompted attempts to heterologously complement the cyanobacterial *ycf51* knockout mutant by expression of the Arabidopsis *Y3IP1* gene. However, these experiments did not lead to functional complementation, suggesting that the 2 proteins either do not have the exact same functions or have coevolved with their interaction partners in a manner that does not permit heterologous complementation.

#### PPD1

PSBP is a PSII subunit involved in photosynthetic oxygen evolution by forming part of the OEC (Yi et al. 2007; Allahverdiyeva et al. 2013). A family of proteins harboring a conserved PSBP region comprises the PSBP-LIKE PROTEINS (PPL) and the PSBP-DOMAIN PROTEINS (PPD; Ifuku et al. 2010; Bricker et al. 2013). PPL1 and PPL2 were described as being involved in PSII repair and NDH complex accumulation, respectively (Ishihara et al. 2007). Later, PPL1 was proposed to participate in PSII supercomplex assembly (Che et al. 2020). The coexpression network of PSBP-related proteins suggested that other family members might also be involved in accumulation of the photosynthetic apparatus and/or related processes (Ifuku et al. 2010). For these reasons, a systematic functional characterization of 6 PPD family members, including PPD1, was conducted by reverse genetics in Arabidopsis (Fig. 4; Table 2; Liu et al. 2012). Loss of *PPD1* expression was found to be lethal upon mutant growth on soil due to the specific loss of PSI. When the abundance of diagnostic PSI subunits in *ppd1* mutants was assessed by immunoblotting, the PsaA-PsaB heterodimer and the subunits PsaC, PSAD, and PSAF were found to be absent. Interestingly, *PPD1* RNAi lines expressing approximately 40% of wild-type PPD1 levels accumulate low amounts of PSI, but no trace of the subunit PSAG (Roose et al. 2014). However, because Arabidopsis *PSAG* mutants suffer only from a moderate reduction in PSI content and photoautotrophic growth is only slightly impaired, the loss of PSAG cannot explain the severe photosynthetic phenotype of the *PPD1* mutants (Jensen et al. 2002; Varotto et al. 2002). Similar to other PSI-deficient mutants, *ppd1* mutants accumulate wild-type levels of LHCA proteins, but LHCI coupling to PSI is impaired (Liu et al. 2012; Roose et al. 2014). Given that PPD1 accumulation decreases in aging leaves while PSI remains stable, PPD1 is unlikely to be required for PSI maintenance (Liu et al. 2012).

Notably, PPD1 is the first described PSI assembly factor localizing to the thylakoid lumen (Fig. 4; Liu et al. 2012). PPD1 has no homolog in cyanobacteria, indicating that its function may be specific to eukaryotic PSI (Fig. 4 and Table 2). Liu et al. (2012) detected interactions of PPD1 with PsaA and PsaB in bimolecular fluorescence complementation (BiFC) assays, but not with the stromal PsaC, the lumenal PSAN, and PSAF. Consistent with its lumenal localization, split-ubiquitin assays revealed that PPD1 binds lumenal loops of PsaA and PsaB (Liu et al. 2012). Interestingly, FLAG-tag–based affinity purification of PPD1-VenusYFP-3xFLAG from a Chlamydomonas overexpression strain followed by tandem mass spectrometric protein identification revealed a set of putative interaction partners, including all known PSI assembly factors except PSA3 (Mackinder et al. 2017). However, PPD1 was not detected in early PSI assembly modules containing the PSI reaction center proteins and the assembly factors Ycf3, Y3IP1, Ycf4, and CGL71/PYG7 in Chlamydomonas (Nellaepalli et al. 2021). In spite of this, and in agreement with the lumenal localization data and the detected protein-protein interactions by BiFC, the *PPD1* RNAi lines display a defect in PSI-donor side electron transfer, and consequently, PPD1 was proposed to mediate PSI reaction center assembly at the lumenal side (Liu et al. 2012; Roose et al. 2014).

#### PSA2

A major advantage of maize over Arabidopsis and tobacco as model to study chloroplast biogenesis lies in the large nutrient reserves in the kernel that permit the soil cultivation of photosynthesis-deficient mutants for an extended period of time. This feature facilitates experiments requiring larger amounts of leaf material, while avoiding secondary effects from heterotrophic in vitro cultivation (Stern et al. 2004; Belcher et al. 2015). A collection of *Mu* transposon-based maize mutants was screened for photosynthetic phenotypes (including low pigment accumulation and high chlorophyll-*a* fluorescence) and led to the establishment of the Photosynthetic Mutant Library (Stern et al. 2004; Williams-Carrier et al. 2010). In this collection, several genes involved in the biogenesis of the photosynthetic apparatus were discovered, including *PHOTOSYSTEM I ASSEMBLY 2* (*PSA2*; Fristedt et al. 2014; Belcher et al. 2015). The *PSA2* ortholog in Chlamydomonas corresponds to *DnaJ-LIKE ZINC-FINGER PROTEIN 1* (*ZNJ1*) and belongs to the PlantCut2 subgroup of the GreenCut2 list. The PlantCut2 set comprises proteins that are conserved in photosynthetic eukaryotes but not prokaryotes (Fig. 4 and Table 2; Karpowicz et al. 2011). The maize and Arabidopsis mutants (*Zm-psa2-1* and *At-psa2-1*) accumulate residual amounts of PSA2 and suffer from a specific loss of PSI accumulation (Fristedt et al. 2014). While the *At-psa2-1* knockdown mutant accumulates low amounts of PSI, permitting its (delayed) development on soil, the *At-psa2-2* and *At-psa2-3* knockout mutants only survive on heterotrophic culture medium (Fristedt et al. 2014; Wang et al. 2016). The currently available evidence argues for a functional relationship between PSA2 and PPD1 in PSI assembly. The 2 proteins are the only described PSI assembly factors of vascular plants that are compartmentalized in the thylakoid lumen (Liu et al. 2012; Fristedt et al. 2014). Additionally, the *Zm-psa2-1* and *At-psa2-1* mutants overaccumulate PPD1, suggesting the potential upregulation of PPD1 to cope with the PSA2 deficiency (Fristedt et al. 2014). Finally, PSA2 and PPD1 co-migrate with a PSAG-containing complex of approximately 250 kDa in native gel electrophoresis (Fristedt et al. 2014), and PSAG accumulation is particularly severely affected in *PPD1* RNAi lines (Roose et al. 2014). However, PSAG but not PPD1 was purified with PSA2 in co-IP assays (Fristedt et al. 2014). Again, because of the much less severe phenotype of Arabidopsis *PSAG* mutants, the interaction of PPD1 and PSA2 with PSAG cannot explain the severe photosynthetic phenotype of the *PSA2* mutants (Jensen et al. 2002; Varotto et al. 2002). Due to its protein disulfide isomerase activity, PSA2 was proposed to promote the formation of disulfide bonds between cysteine residues at the lumenal side of PSI, which could stabilize the PSI subunits and possibly aid the transition towards the final steps of PSI assembly (Fristedt et al. 2014).

#### PSA3

The PSI assembly factor PHOTOSYSTEM I ASSEMBLY 3 (PSA3) was identified by a mutant screen in Arabidopsis (Fig. 4; Table 2; Shen et al. 2017). In the Chloroplast Function Database II, which contains a collection of Arabidopsis mutants for nucleus-encoded plastid proteins, a *psa3* T-DNA insertion mutant line was found and reported to display a pale green phenotype (Myouga et al. 2013). Similar to PSA2, the maize homolog of PSA3 was later identified in the Photosynthetic Mutant Library (Fristedt et al. 2014; Belcher et al. 2015; Shen et al. 2017). While the maize mutant *Zm-psa3* suffers from complete loss of PSI accumulation, the Arabidopsis mutant *At-psa3* accumulates residual amounts of PSI that, however, are not sufficient for survival on soil (Shen et al. 2017). In maize, PSA3 accumulation is independent of PSA2, Y3IP1, and Ycf3 accumulation but is slightly reduced in the PYG7-deficient mutant (Shen et al. 2017). PSA3 interacts with PYG7, and both proteins associate with a similar PSI subcomplex that is slightly smaller than the mature PSI-LHCI in BN-PAGE (Shen et al. 2017). This subcomplex is also decreased in *Zm-psa3* and *Zm-pyg7*, and the PSA3 association with this complex is reduced in *Zm-pyg7* (Shen et al. 2017). Finally, both PYG7 and PSA3 interact with PsaC (Shen et al. 2017; Yang et al. 2017). Therefore, PSA3 likely has a cooperative role during the assembly of PSI-LHCI, presumably by mediating the attachment of PsaC to the PSI reaction center and/or protecting the stromal side of non-functional PSI intermediates (Heinnickel et al. 2016; Shen et al. 2017; Yang et al. 2017; Nellaepalli et al. 2021). In line with such a role, the redox state of the 2 cysteines C199 and C200 appears to be important for PSA3 function in that elevated amounts of the reduced form of PSA3 are observed in conditions triggering PSI photodamage (Chen et al. 2022b). Since PYG7/Ycf37 but not PSA3 is conserved in cyanobacteria, this collaborative function of PYG7 and PSA3 has likely evolved with eukaryotic PSI (Fig. 4 and Table 2; Heinnickel et al. 2016; Shen et al. 2017).

#### CEPA1

Given the required cooperation between the PSI assembly factors, they can be expected to be coexpressed during PSI biogenesis. The EnsembleNet tool generates lists of genes that are predicted to be functionally related to a set of genes of interest in Arabidopsis (largely) based on integrated coexpression data (Hansen et al. 2018). The EnsembleNet search using as query the 5 nucleus-encoded PSI assembly factors PYG7, Y3IP1, PPD1, PSA2, and PSA3 yielded a list of genes that may contribute to PSI biogenesis. Among them, genes coding for plastid-localized proteins with uncharacterized function were considered as potential candidates for new PSI assembly factors. The corresponding knockout mutants were then subjected to a so-called qL screening, in which the redox state of the PSII acceptor side (qL parameter; Kramer et al. 2004) at low light intensities was determined by pulse-amplitude modulation (PAM) chlorophyll-*a* measurements in young seedlings. Because a defect in PSI accumulation results in imbalanced excitation rates of the 2 photosystems, the PSII acceptor side of PSI mutants becomes rapidly reduced in low light, resulting in a strong decrease of qL. This screen identified the gene *CO-EXPRESSED WITH PHOTOSYSTEM I ASSEMBLY 1* (*CEPA1*; Fig. 4 and Table 2; Rolo et al. 2024). The *cepa1-3* mutant, which completely lacks the CEPA1 protein, suffers from a strongly reduced PSII acceptor side already at low light intensities, resulting in retarded growth and light green leaves. This phenotype was shown to arise from a strong decrease in PSI accumulation (to only 25% of wild-type amounts), while the other thylakoidal protein complexes are largely unaffected.

In native gels, CEPA1 comigrates with several PSI subcomplexes and the assembly intermediate PSI*. CEPA1 also comigrates with a PSI subcomplex that is slightly smaller than the mature PSI-LHCI, which is decreased in the *cepa1-3* mutant and may be the complex that Ycf4, PSA3, and PYG7 were previously suggested to comigrate with (Krech et al. 2012; Shen et al. 2017; Yang et al. 2017). Interestingly, CEPA1 directly interacts with PSA3 and may be part of a complex containing PPD1 (Rolo et al. 2024). PSA3 was previously shown to interact with PYG7 (Shen et al. 2017), but the precise relationship between these 3 PSI assembly factors is currently not yet clear. The absence of CEPA1 results in less severe defects than that of PSA3 (and PYG7) in Arabidopsis, as null mutants survive on soil and no specific PSI subunit(s) appear to be missing (Stöckel et al. 2006; Shen et al. 2017; Yang et al. 2017; Rolo et al. 2024). It therefore seems possible that CEPA1 affects the stability (e.g. by anchoring or clearing) of PSA3, either in association or in competition with PYG7. This possibility clearly warrants further investigation. CEPA1 was also identified by Zhang et al. (2024) in a screen for photosynthesis mutants, and the protein was named PHOTOSYSTEM I BIOGENESIS FACTOR 8 (PBF8). While PSA3 and PYG7 interact with PsaC, CEPA1/PBF8 was shown to interact with the PSI reaction center PsaA-PsaB, but also PSAE and PSAF (Zhang et al. 2024), further supporting a cooperative role of CEPA1 and PSA3 in mediating stromal ridge incorporation. Because CEPA1/PBF8 seems to associates with PSI* to form an assembly intermediate (Rolo et al. 2024; Zhang et al. 2024), it could also promote the incorporation of PSAF, thus triggering the maturation of the assembly intermediate into the mature complex.

As mature CEPA1 associates with PSI-related (sub)complexes, while abundant CEPA1 fragments were detected in the free protein fraction in BN-PAGE, it was suggested that CEPA1 degradation could be directly related to its function, for example, by proteolytic cleavage detaching CEPA1 from PSI assembly complexes after having fulfilled its function (Rolo et al. 2024). A knockdown mutant, *cepa1-1*, accumulates approximately one-half of CEPA1 wild-type levels. This reduction in CEPA1 protein abundance causes no measurable defect in any phenotypic parameter, indicating that CEPA1 may be present in excess and does not limit PSI assembly.

Interestingly, CEPA1 is absent from cyanobacteria, but a putative ortholog is present in Chlamydomonas, albeit the protein sequence displays a relatively low conservation (Rolo et al. 2024). This could explain why studies reporting the Chlamydomonas protein did not indicate any Arabidopsis homolog (Mackinder et al. 2017; Li et al. 2019b; Fauser et al. 2022; Kafri et al. 2023), and vice versa (Zhang et al. 2024). The putative CEPA1 homolog was co-immunopurified with PPD1 in Chlamydomonas (Mackinder et al. 2017), in line with PPD1 associating with a CEPA1-containing complex in Arabidopsis, even though no direct physical interaction has been detected (Rolo et al. 2024). Large-scale screens of Chlamydomonas mutants had revealed a photosynthetic defect in strains whose *CEPA1* homolog was disrupted, and therefore, this gene was preliminarily annotated as *LIGHT GROWTH SENSITIVE 1* (*LGS1*; Table 2; Li et al. 2019b; Fauser et al. 2022). Recently, it was shown that the mutant suffers from a specific PSI subunit decrease, a finding that motivated the authors to rename this gene *PHOTOSYSTEM I REQUIRED 1* (*PIR1*; Table 2; Kafri et al. 2023). Although currently no detailed description of the mode of action of PIR1 is available, it seems that *CEPA1* disruption in Arabidopsis causes a less severe phenotype than that of its putative ortholog *PIR1* in Chlamydomonas, given that *pir1* knockout strains display little to no growth in photoautotrophic conditions (Kafri et al. 2023). A thorough comparison of CEPA1 function in land plants and PIR1 function in microalgae will be of particular interest to better understand how this assembly factor coevolved with eukaryotic PSI.

#### RMT2

Screening of the Chlamydomonas mutant library revealed several uncharacterized genes linked to photosynthetic processes, including potential PSI assembly factors (Li et al. 2019b; Fauser et al. 2022; Kafri et al. 2023). Similar to PIR1, a mutant lacking RuBisCO METHYLTRANSFERASE 2 (RMT2) suffered from a specific PSI subunit depletion (Kafri et al. 2023). Subsequently, RMT2 was characterized in more detail after *rmt2* mutants had been selected as strains highly sensitive to O_2_ (Kim et al. 2024). Despite its annotation, RMT2 does not seem to be involved in RuBisCO methylation, but instead, may participate in PSI accumulation at the post-translational level in Chlamydomonas (Kim et al. 2024). The levels of the PSI assembly factors Ycf3 and Ycf4 are increased in *rmt2*, potentially as a response to reduced PSI accumulation. Interestingly, the PSI subunit PsaB, the LHCI subunit LHCA4, and the PSI assembly factor PIR1 were enriched in proximity labeling experiments using a biotin ligase-tagged RMT2 as a bait (Kim et al. 2024). Also, RMT2 was previously co-purified with the tagged-PPD1 (Mackinder et al. 2017). Exploring whether RMT2 directly interacts with these proteins and elucidating the underlying functional relationships would help to pinpoint the exact role of RMT2.

Although RMT2 has some of the features conserved in methyltransferase proteins, the currently available data do not suggest any difference in PSI subunit methylation in the absence of RMT2 (Kim et al. 2024). Thus, whether RMT2 mediates PSI assembly via a potential methyltransferase activity remains to be elucidated. The closest homolog of RMT2 in Arabidopsis is LYSINE METHYLTRANSFERASE-LIKE (LSMT-L; encoded by At1g14030; Kim et al. 2024). The knockout mutant *lsmt1* was reported to not show a developmental defect (Mininno et al. 2012), as opposed to PSI assembly factor mutants. Thus, further work is needed to determine LSMT-L function and clarify its potential role in PSI assembly.

#### FKB20-2

The immunophilin FKB20-2 is localized in the thylakoid lumen of Chlamydomonas chloroplasts. Immunophilins are cis-trans peptidyl-prolyl isomerases that assist in the folding of their client proteins. Chlamydomonas *fkb20-2* mutants suffer from a 50% reduction in PSI core subunit accumulation, while accumulation of PSII and the other photosynthetic complexes is unaltered. When grown in high light, ROS production of *fkb20-2* is strongly increased, resulting in bleaching of the mutant cultures. A HA-tagged version of FKB20-2 complemented the mutant phenotype and co-purified with PSAG (Guo et al. 2024). Interestingly, the other 2 PSI assembly factors localized in the thylakoid lumen, PSA2 and PPD1, co-migrate with a PSAG-containing complex, and PSAG co-purified with PSA2 (Fristedt et al. 2014), possibly suggesting a joint function with FKB20-2. Mutants of the FKB20-2 homolog in Arabidopsis, AtFKBP20-2, suffer from decreased accumulation of PSII-LHCII supercomplexes, while the relative abundance of dimeric PSII is increased (as evidenced by native gel electrophoresis). AtFKBP20-2 was therefore suggested to support PSII supercomplex formation (Lima et al. 2006). Despite a high degree of sequence conservation between CrFKB20-2 and AtFKBP20-2, the high light sensitivity of the Chlamydomonas *fkb20-2* mutant could not be rescued by heterologous complementation with *Atfkbp20* (Guo et al. 2024). Therefore, both homologs seem to have adopted different functions and/or undergone evolutionary changes in their client proteins. Client switches could potentially explain why PSI biogenesis is most sensitive to FKB20-2 loss in Chlamydomonas, while PSII supercomplex formation is most strongly affected in vascular plants. However, it cannot fully be excluded that AtFKBP20-2 is a genuine PSI assembly factor, because photosynthetic complex accumulation was not assessed by Lima et al. (2006). Arabidopsis mutants suffering from a moderate reduction in PSI content such as the *cepa1* mutant show a massive over-reduction of the PSII acceptor side, especially in low light, which was the basis of the qL screen for mutants with decreased PSI accumulation (Rolo et al. 2024). Such an over-reduction of the PSII acceptor side and the PQH pool results in STN7 kinase activation and induces transition to state 2, which potentially could explain the decreased PSII supercomplex formation observed in the *Atfkbp20-2* mutant. In the light of these considerations, a detailed re-investigation of the *Atfkbp20-2* mutant, including a precise analysis of photosynthetic complex accumulation and state transitions, might be warranted.

#### Cyanobacteria-specific assembly factors for PSI

As discussed above, some PSI assembly factors are absent from cyanobacteria and were evolutionarily acquired in photosynthetic eukaryotes. Previously, the protein BtpA was proposed to be a PSI assembly factor present only in prokaryotes. BtpA was identified in a screen of photosynthesis-deficient Synechocystis mutants (Bartsevich and Pakrasi 1995, 1997; Zak and Pakrasi 2000). Recently, the function of BtpA was redefined by showing that both photosystems are nearly undetectable in the absence of BtpA, presumably due to impaired chlorophyll biosynthesis (Skotnicová et al. 2024). Intriguingly, increasing the amounts and/or the stability of GluTR (glutamyl-tRNA reductase), an early enzyme of the tetrapyrrole biosynthetic pathway (that provides the precursors of chlorophyll and heme) in the *btpA* knockout background restored photoautotrophy. BtpA interacts with and stabilizes GluTR, suggesting a role in the regulation of chlorophyll biosynthesis rather than in PSI assembly (Skotnicová et al. 2024).

Very recently, a novel PSI assembly factor, Ycf51, was identified in cyanobacteria but seems to have disappeared from the eukaryotic lineages of photosynthetic organisms (except for glaucophyte algae and the photosynthetic amoeba *Paulinella*; Table 2; Dai et al. 2024). Ycf51 was isolated through the systematic characterization of *ycf* genes with unknown function (Dai et al. 2024). Ycf51 participates in PSI assembly by interacting with PsaC and Ycf3 but is not required for anchoring Ycf3 to thylakoid membranes. As opposed to the Δ*ycf3* knockout, which completely lacks PSI (Schwabe 2003), *ycf51* knockout strains still accumulate small amounts of PSI that are sufficient for (reduced) photoautotrophic growth. As Ycf51 is absent from green algae and land plants, which have acquired Y3IP1 (Table 2), it seemed reasonable to speculate that the Ycf51 function was taken over by Y3IP1, given that both proteins interact with Ycf3 (Albus et al. 2010; Nellaepalli et al. 2018; Dai et al. 2024). However, the sequence divergence of Ycf51 and Y3IP1, and the inability of the Arabidopsis *Y3IP1* gene to complement the Synechocystis *ycf51* knockout mutant, suggest that Ycf51 is not the missing ancestor of Y3IP1 in photosynthetic prokaryotes (Dai et al. 2024).

Because of its topology (i.e. insertion into the thylakoid membrane via 2 transmembrane domains, with a stromal soluble C terminus) and because it interacts with PsaC, Ycf51 may participate in stromal ridge assembly and/or protection (Dai et al. 2024). The PSI assembly factor Ycf37, which also participates in stromal ridge formation, notably by interacting with PsaC (Dühring et al. 2006), was not reported in the list of proteins pulled down with Ycf51 (Dai et al. 2024). A possible (partial) redundancy of the 2 proteins could be investigated by assessing whether overexpression of *ycf37* would at least partially complement the *ycf51* knockout phenotype and vice versa.

Interestingly, *ycf51* is part of the *ycf51*(*sll1702*)-*sppa1*(*sll1703*)-*csgA*(*sll1704*) transcription unit (Kaneko et al. 1996; Dai et al. 2024). Sppa1 is a protease involved in high light acclimation, but probably not specifically in PSI biogenesis (Lensch et al. 2001; Pojidaeva et al. 2004; Wetzel et al. 2009; Dai et al. 2024). Nonetheless, in view of the functional links between PSI accumulation and light acclimation (Chen et al. 2022b), it may be worthwhile to investigate a possible role of Sppa1 in PSI turnover. However, there is currently no evidence of substantial degradation of PSI during high-light acclimation, given that, at least in most land plants, PSI contents are largely unaffected by changes in growth light intensity (Chow and Anderson 1987; Chow and Hope 1987; Schöttler et al. 2017; reviewed by Schöttler and Tóth 2014).

## Challenges with resolving the molecular details of the PSI assembly pathway

### Integration of data on PSI assembly from different photosynthetic lineages

The PSI reaction center is remarkably conserved from cyanobacteria to land plants (Amunts and Nelson 2008, 2009). Nonetheless, there are significant differences in PSI structure and function between the different lineages of photosynthetic organisms. First, PSI is present as a monomer in land plants (Caspy et al. 2020) but (predominantly) exists as a trimer in cyanobacteria (Jordan et al. 2001; Chen et al. 2022a). Additionally, 2 PSI core subunits present in cyanobacteria have been lost in vascular plants during evolution: the cyanobacteria-specific protein PsaX (Jordan et al. 2001), and PsaM, which is also present in the moss *Physcomitrium patens* (Yan et al. 2021) as well as in red algae (Pi et al. 2018) and in the marine green macroalga *Bryopsis corticulans*, but not in the unicellular green alga Chlamydomonas (Qin et al. 2019). Other subunits have newly appeared in eukaryotes: PSAG, PSAH, PSAN, and PSAO (Amunts and Nelson 2009; Yang et al. 2015). Also, the essentiality and function of some conserved subunits have changed in evolution. For example, complete loss of PSAD allows accumulation of PSI in cyanobacteria but not in Arabidopsis (Xu et al. 1994; Ihnatowicz et al. 2004), and PSAL participates in PSI trimerization in cyanobacteria but is important for LHCII docking during state transitions in land plants (Grotjohann and Fromme 2005; Yang et al. 2015; Pan et al. 2018; Li et al. 2019a). Moreover, eukaryotic PSI has gained the LHC, suggesting the requirement for additional mechanisms to build the mature PSI-LHCI complex (Amunts and Nelson 2009). In addition to these structural and functional differences, PSI assembly also had to adapt to new environmental challenges. First, the cellular environment changed by the ancestral cyanobacterial endosymbiont becoming integrated into the metabolism and genetic networks of the eukaryotic cell and additionally by the shift from unicellularity to multicellularity. Second, the colonization of land habitats (terrestrialization) led to new environmental challenges, including exposure to drought, high light intensities, and UV light. This is reflected, for example, by a change in PSI antenna size. Whereas LHCI is formed by 10 LHCAs (LHCA2-9 and 2 LHCA1) in Chlamydomonas (Ozawa et al. 2018; Su et al. 2019), it is composed of only 4 LHCAs (LHCA1-4 in the PSI-LHCI supercomplex) in land plants, and the concomitant reduction in antenna cross-section likely represents an evolutionary adaptation to terrestrial environments with higher light intensities.

Gene structure, organization, and expression have also undergone major evolutionary changes. Genes for several PSI subunits and assembly factors were transferred to the nuclear genome in photosynthetic eukaryotes. In addition, genes retained in the chloroplast genomes changed their structure and arrangement in operons. For example, in land plants, *psaA* and *psaB* are part of the same transcription unit (*psaA-psaB-rps14* operon), *psaI* is a plastid gene, *ycf3* is located downstream of the *psaA*-*psaB*-*rps14* operon, and *ycf4* is part of the *psaI*-*ycf4*-*cemA*(*ycf10*)-*petA* gene cluster (Shinozaki et al. 1986; Ruf et al. 1997; Krech et al. 2012; Schöttler et al. 2017). By contrast, in Chlamydomonas, the *psaA* gene is split into 3 discontinuous exons in the plastid genome that are joined at the mRNA level by trans-splicing and are physically distant from the *psaB* gene. Also, *psaI* was transferred to the nuclear genome, and *ycf3* and *ycf4* are part of the same transcription unit (*rps18*-*ycf3*-*ycf4*-*rps9;* Kück et al. 1987; Boudreau et al. 1997; Maul et al. 2002; Merchant et al. 2007). Hence, the regulation of PSI subunit supply and the early steps of the assembly process may differ between green algae and land plants.

In view of these substantial differences in PSI structure, function, and biosynthesis, caution needs to be exercised before generalizing assembly mechanisms that were worked out in a single lineage of photosynthetic organisms. Although there is accumulating evidence for evolutionary conservation of a substantial part of the PSI assembly process from cyanobacteria to seed plants, findings made in one of the 3 major model systems (cyanobacteria, Chlamydomonas, seed plants) should be experimentally validated in the 2 other systems.

### Taking snapshots of PSI assembly steps

Based on the stepwise PSI-LHCI assembly model, intermediates containing various subsets of PSI-LHCI subunits should be found along the PSI-LHCI assembly pathway (Fig. 3). The characterization of such intermediate complexes can be very informative in that it can reveal the order in which the subunits are inserted into the growing complex. Postulated intermediate PSI complexes are represented in Fig. 3, and their theoretical molecular masses are given, although one would expect that association of PSI assembly factors with each of these complexes increases their molecular weight and potentially affects their conformation and 3-dimensional structure. Some assembly intermediates may even be bigger than the mature PSI-LHCI, as exemplified by the isolation of a Ycf4-PSI assembly complex (of >1,500 kDa) that is more than twice the size of the mature PSI-LHCI (750 kDa) in Chlamydomonas (Ozawa et al. 2009; Su et al. 2019). However, capturing genuine assembly intermediates remains a challenging undertaking for both biological and technical reasons, and to date, only few bona fide PSI assembly intermediates have been characterized (Dühring et al. 2007; Ozawa et al. 2010; Wittenberg et al. 2017).

### The assembly of PSI is fast

A significant challenge associated with the identification of assembly intermediates is posed by the biogenesis of PSI-LHCI being a relatively fast process (Ozawa et al. 2010; Wittenberg et al. 2017). Ozawa et al. (2010) conducted a pulse-chase labeling experiment in Chlamydomonas, to follow newly synthesized subunit accumulation and insertion into complexes. They observed that the late PSI intermediate lacking PSAG and PSAK but containing loosely associating LHCI was synthesized de novo within 1 minute. This makes it clear that most assembly intermediates are short-lived and accumulate only transiently and to very low levels. It might be worthwhile to test if PSI assembly can be slowed down by adverse conditions, especially cold stress. While cold stress lasting for several hours to multiple days, depending on the species, can result in PSI photoinhibition (Zhang and Scheller 2004; Rogalski et al. 2008; Sonoike 2011), short-term treatments may result in the enrichment of assembly intermediates in developing leaves.

As opposed to the fast PSI assembly during thylakoid membrane biogenesis, PSI recovery after photoinhibition is very slow. PSI-LHCI photoinhibition can occur in stressful conditions that lead to excess electron accumulation at the PSI acceptor side, giving rise to superoxide radical production at the [Fe_4_–S_4_] clusters (Sonoike 2011). For example, chilling stress massively slows down the consumption of NADPH by the Calvin–Benson–Bassham cycle, while photosynthetic electron transport is less affected by temperature changes. Chilling stress, therefore, induces over-reduction of the PSI acceptor side and triggers PSI-LHCI photoinhibition, followed by degradation of damaged PSI core proteins (Zhang and Scheller 2004). In Arabidopsis, PSI core accumulation was still decreased after a week of recovery at normal temperature, while PSII contents had fully recovered from photoinhibition within 8 h (Zhang and Scheller 2004). A thorough comparison of the PSI assembly processes during thylakoid membrane biogenesis vs recovery from photoinhibition could be very informative and could help with pinpointing limiting factors such as subunit provision or assembly factor abundance and may even reveal new assembly intermediates. However, in contrast to PSII, damaged PSI is thought to be reassembled de novo (Zhang and Scheller 2004), although some free PSI subunits such as PSAD, PSAE, PSAG, and PSAK can spontaneously replace their counterparts in fully assembled PSI-LHCI complexes in vitro, without requiring disassembly of the complex (Mant et al. 2001; Minai et al. 2001; Lushy et al. 2002; Zygadlo et al. 2006). Whether subunit replacement also occurs in vivo and provides a repair pathway that bypasses the requirement to rebuild the whole complex and degrade the damaged PSI remains to be determined.

As the PSII deassembly/reassembly cycle is important for photosynthesis regulation, PSII turnover is relatively high (Li et al. 2018), thus potentially leading to the accumulation of PSII intermediates when the cycle is out of balance. By contrast, once assembled, PSI-LHCI is relatively stable and its turnover is low (Nelson et al. 2014; Li et al. 2018). Additionally, PSI-LHCI content does not decrease in senescing leaves except in cereal species (reviewed by Schöttler and Tóth 2014), with the possible reason being that the photosynthetic apparatus represents a large sink of nitrogen that needs to be tapped to facilitate grain filling (Belcher et al. 2015). Because PSI-LHCI is stable in aging leaves and has a low turnover, PSI-LHCI assembly is thought to occur nearly exclusively in young developing leaves. In line with this model, PSI assembly factors are mostly expressed in developing leaf tissues (Krech et al. 2012; Klepikova et al. 2016; Rolo et al. 2024). Overall, because PSI-LHCI assembles quickly and mostly in developing leaves, has a low turnover, and is stable over time, most PSI intermediates are expected to be short-lived and lowly abundant, which, at least in part, explains the lack of resolution in the current PSI-LHCI assembly model. The analysis of young greening tissue can facilitate the detection of assembly intermediates, as suggested by the isolation of substantial amounts of PSI* from very young tobacco leaves (Wittenberg et al. 2017).

### Catching PSI assembly intermediates

In addition to the intrinsic properties of the PSI-LHCI assembly process, the methodology involved in detecting and characterizing assembly intermediates is also challenging. In theory, slowing down the assembly process and, in this way, enriching complex intermediates could be achieved by employing appropriate mutants that lack a subunit of the complex or are deficient in a specific assembly factor. However, in the case of PSI-LHCI biogenesis, this strategy is not straightforward, because loss of many subunits, including PsaA, PsaB, PsaC, and PSAD, completely abolishes PSI-LHCI assembly. By contrast, loss of several non-essential subunits such as PsaI leads to synthesis of a full PSI-LHCI complex that lacks only that single subunit, but does not disturb PSI accumulation (Schöttler et al. 2017). An advantage of using cyanobacteria is that PSI mutant phenotypes are often less severe than in land plants (Xu et al. 1994; Wilde et al. 1995, 2001; Dühring et al. 2006), which can help with detecting stalled PSI assembly intermediates (Dühring et al. 2007). The use of knockdown and inducible knockout lines provides a promising alternative in land plants, as exemplified by an inducible *PSAF* RNAi line in tobacco that over-accumulated PSI* upon induction (Wittenberg et al. 2017).

As mentioned above, the PsaA–PsaB heterodimer accounts for more than one-half of the size of the PSI core, and the other PSI core subunits are rather small (Fig. 2A and Table 1). Therefore, when using classic complex separation methods based on size (e.g. BN-PAGE or sucrose density gradients), it becomes challenging to resolve PSI subcomplexes (Fig. 3). Once isolated, the complete characterization of intermediate complexes and their subunit composition poses new challenges. The solubilization methods required to isolate highly hydrophobic membrane-embedded complexes can induce the dissociation of loosely associated subunits which may occur in assembly intermediates (Ozawa et al. 2010). Downstream mass spectrometric protein identification often suffers from limited detection sensitivity of hydrophobic peptides, making the reliable identification of small membrane proteins like PsaI and PsaJ difficult. As small membrane proteins are also poor antigens, detection by immunoblotting does not provide a viable alternative. Identification of small subunits by cross-linking mass spectrometry may be a worthwhile approach to pursue in future studies.

Taken together, an unfortunate combination of intrinsic biological constraints and technical difficulties have hampered progress with elucidating the PSI-LHCI assembly process in molecular detail and are chiefly responsible for the lack of sufficient resolution in our current models of the assembly pathway.

To avoid artifacts arising from their low stability during thylakoid isolation and solubilization, spectroscopic methods can also be used to better characterize at least some PSI assembly intermediates. For example, functional photosynthetic parameters measured in assembly factor mutants that exhibit overaccumulation of a specific assembly intermediate can be compared with the defects in mutants of the different non-essential subunits. This approach may become particularly useful when the small differences in molecular mass that come from the addition of low molecular mass subunits to the reaction center core cannot be resolved by size-dependent separation techniques but have a clear impact on the function of PSI. Especially the functions of the PSI donor side (i.e. plastocyanin oxidation) and the PSI acceptor side (i.e. ferredoxin reduction) can be probed with biophysical approaches. Simple, indirect measurements include light response curves of the yield of P_700_, because an increased donor side limitation of P_700_ (i.e. Y(ND), the accumulation of P_700_^+^) under light-limited conditions might indicate inefficient reduction of P_700_^+^ by plastocyanin. On the other hand, an increased acceptor-side limitation of PSI (Y(NA)), occurring especially at high light intensities and high flux rates of LEF, can indicate impaired forward electron transfer to ferredoxin and the FNR. More advanced spectroscopic approaches allow the direct measurement of absorbance changes of plastocyanin and ferredoxin, in parallel with absorbance changes of P_700_ (Klughammer and Schreiber 2016; Schreiber and Klughammer 2016). This enables direct measurements of redox equilibration between reduced plastocyanin and P_700_^+^, or between reduced P_700_ and oxidized ferredoxin, to obtain quantitative parameters of the functionality of different enriched PSI assembly intermediates. By comparing these changes with those observed in single subunit mutants with defects in these processes, it will become possible to distinguish between PSI (sub)complexes of similar size (Schöttler et al. 2007; Wittenberg et al. 2017). If an over-accumulating assembly intermediate is impaired in reactions at either its donor or its acceptor side, this should result in a clear heterogeneity in PSI redox equilibration with plastocyanin or ferredoxin, respectively. By fitting of the redox equilibration signals, it should be possible to deconvolute the total signal into equilibration constants similar to wild-type plants (for the mature PSI population) and similar to mutants with impaired plastocyanin oxidation (such as the *PSAF* antisense mutants; Wittenberg et al. 2017) or ferredoxin reduction.

### PSI degradation

Little, if anything, is known about regulated PSI degradation in response to changing growth conditions and/or during leaf ontogenesis. This is somewhat surprising, given that chloroplast proteases have been studied for a long time and in great detail, and proteases that specifically act in the degradation of the other major photosynthetic complexes have been successfully identified. While the ClpP protease predominantly degrades stromal proteins including RuBisCO (Majeran et al. 2019), PSII degradation during the PSII repair cycle is catalyzed by several cooperating proteases, including the thylakoid-associated FtsH proteases and Deg proteases (reviewed by Su et al. 2024). In Chlamydomonas, rapid degradation of Cyt*b*_6_*f* under sulfur or nitrogen deprivation is also catalyzed by the FtsH protease (Malnoë et al. 2014; Wei et al. 2014).

It seems unlikely that the FtsH protease plays a role in PSI degradation. When Arabidopsis mutants deficient in either the FTSH2 or the FTSH5 subunit, resulting in reduced FtsH activity, were grown in moderate light (so that the PSII repair cycle was unaffected), they suffered from a 50% reduction in PSI accumulation. However, this was not due to altered PSI degradation rates. Instead, FtsH protease repression resulted in impaired biogenesis of PSI. While neither mRNA stability nor translation initiation of *psaA* or *psaB* were impaired in the mutants, the synthesis rates of both PsaA and PsaB were decreased to 30% of wild-type levels. The reason for this reduced synthesis of the PSI reaction center core subunits could not be elucidated. Remarkably, accumulation of Y3IP1 and Ycf4 was strongly increased in the 2 mutants, possibly as a compensatory response (Järvi et al. 2016).

The identification of proteases involved in the degradation of PSII and Cyt*b*_6_*f* has strongly benefited from the identification of conditions triggering high degradation rates such as PSII photoinhibition, or sulfur and nitrogen starvation in Chlamydomonas. For PSI, at least 3 conditions are known to result in markedly increased rates of PSI degradation, and therefore, could provide promising entry points into the identification of PSI proteases. During chilling-induced PSI photoinhibition, distinct degradation intermediates of both PsaA and PsaB were observed in barley, possibly indicating a complex series of proteolytic cleavage steps. For PsaB, 7 distinct degradation fragments could be resolved by immunoblotting (Tjus et al. 1999). However, because the responsible protease has not been identified so far, it cannot be excluded that these degradation intermediates arise from oxidative cleavage of the protein. Alternatively, PsaA and PsaB could undergo conformational changes after photo-oxidative modifications of specific amino acid residues, which then could make them accessible to proteolytic cleavage, similar to the situation described for the D1 protein in PSII repair (Kato et al. 2023).

Besides PSI photoinhibition, leaf senescence is another obvious condition to search for PSI proteases. With the notable exception of cereals, which have to remobilize nitrogen from the leaf for grain filling, in most angiosperms, PSI contents remain constant until the very last phases of leaf senescence, when rapid PSI degradation sets in (reviewed by Schöttler and Tóth 2014). Because the contents of PSII, Cyt*b*_6_*f*, and the chloroplast ATPase start to decline much earlier during leaf development (Schöttler and Tóth 2014), it seems unlikely that the same proteases involved in their degradation also target PSI. Interestingly, in the Δ*psaI* mutant, loss of PSI was accelerated during leaf senescence, while PSI accumulation was unaltered in earlier developmental stages of the leaf, preliminarily suggesting that, during early senescence, a protease is induced that breaks down mutant PSI much more readily than wild-type PSI complexes (Schöttler et al. 2017). This could be a proteolytic activity that primarily targets one of the other photosynthetic complexes.

Rapid degradation of PSI has also been observed when plants are exposed to major changes in growth light quality. When grown under artificial light regimes preferentially exciting PSI (“PSI light,” enriched in far-red light), PSI contents are much lower than under light regimes preferentially exciting PSII (“PSII light”). In response to irradiation with PSII light, plants increase PSI accumulation to compensate for imbalanced excitation of the photosystems. When plants are moved from “PSII light” to “PSI light,” pea (*Pisum sativum*) plants respond by degrading up to 35% of their PSI complexes within 3 days, while new PSII is synthesized (Kim et al. 1993). Similar, but smaller, changes in PSI and PSII accumulation can also be induced by light shifts in Arabidopsis (Dietzel et al. 2011). Therefore, also light quality shifts could be used as an experimental system to specifically induce PSI degradation and identify the responsible proteases.

Finally, because PSI degradation during senescence and upon light shifts is a process occurring at a time scale of several days, it is also conceivable that proteases target the PSI assembly factors instead of PSI itself. Although PSI is exceptionally stable, and degradation rates below 0.1 per day have been calculated for most of its subunits, the 2 reaction center subunits PsaA and PsaB showed somewhat higher degradation rates of up to 0.2 per day, indicating that some residual de novo biogenesis of PSI may occur in mature leaves (Li et al. 2017, 2018). Therefore, complete abolishment of the de novo biogenesis (*e.g.* by degradation of the PSI assembly machinery and/or repression of the genes encoding it) could also explain the decline in PSI observed upon light shifts and during leaf senescence (and would also explain why, so far, no specific PSI protease has been identified). Recently, in cyanobacteria, the FtsH4 protease was shown to degrade the PSI assembly factors Ycf4 and Ycf37, the homolog of PYG7, as well as unassembled PsaA and PsaB (Koník et al. 2024). These observations would be consistent with a model, in which PSI levels are mainly adjusted through controlled changes in assembly factor abundance.

## Concluding remarks and future perspectives

Over the past 3 decades, the determination of high-resolution 3-dimensional structures of PSI complexes and the functional characterization of PSI subunits, together with the identification of PSI assembly intermediates and the discovery of PSI assembly factors, have greatly enhanced our understanding of PSI biogenesis. However, we are still far from a comprehensive understanding of the PSI assembly process and lack a complete parts list of the assembly machinery.

Several approaches were successfully applied to identify PSI assembly factors, including the functional characterization of plastid ORFs (Ycf3 and Ycf4; Wilde et al. 1995; Boudreau et al. 1997; Ruf et al. 1997; Krech et al. 2012), searches for conserved domains potentially related to photosynthetic complex assembly (PYG7 and PPD1; Wilde et al. 2001; Liu et al. 2012), screens for proteins interacting with known PSI assembly factors (Y3IP1; Albus et al. 2010), unbiased screening of mutant collections with defects in photosynthesis (PSA2 and PSA3; Fristedt et al. 2014; Shen et al. 2017), and functional prediction based on integrated coexpression networks (CEPA1; Rolo et al. 2024).

Both biochemical and bioinformatic approaches will likely remain relevant to identify the PSI assembly factors that are still missing. For example, co-immunoprecipitation experiments with tagged PSI assembly factors have not yet been exhaustively employed, and the continuous improvement of proximity labeling techniques bodes well for the elucidation of interaction networks involved in PSI assembly (e.g. Feng et al. 2024). Also, the rapidly progressing development of algorithms for modeling and predicting protein–protein interactions, further accelerated by the exploitation of artificial intelligence–based approaches, including the AlphaFold structure prediction software (Evans et al. 2021; Jumper et al. 2021; Bryant et al. 2022; Liu et al. 2023), represents a powerful discovery tool for new PSI assembly factors and PSI assembly modules in the near future. In combination with cross-linking mass spectrometry, improved structural predictions should allow us to determine the contact points between assembly factors and their client PSI subunits at high resolution, thus revealing the exact mode of action of the PSI assembly machinery (Dai et al. 2024).

Many of the above-discussed approaches can potentially yield large lists of candidate genes, thus requiring rapid screening methods for the corresponding mutants. In this regard, the qL screening proved to be a fast and relatively easy method to preliminarily identify mutants that are affected in PSI accumulation (Rolo et al. 2024). Arabidopsis mutants can be grown in vitro, and screening can be conducted a week after germination, with little labor involved. Genes relevant to other photosynthesis-related functions can escape detection by the qL screening, but they could be revealed by measuring other chlorophyll-*a* fluorescence parameters by PAM fluorometry (Rühle et al. 2018).

Finally, it should be borne in mind that loss of information is almost unavoidably associated with the isolation of PSI complexes and assembly intermediates. It will be important to ultimately integrate the PSI assembly mechanisms into their physiological context. For example, subunit assembly and cofactor attachment are mostly studied independently, despite being intimately linked and highly coordinated to facilitate proper protein folding and complex formation in vivo. Exploring the relationships between the many interconnected processes contributing to PSI biogenesis and resolving the underlying physical interactions and regulatory networks will become an increasingly important task. We can also expect that advances in structural analyses by cryo-electron tomography will soon provide us with exciting insights in the assembly processes as they occur in their native cellular environment and allow us to contextualize individual assembly steps and mechanisms (Turk and Baumeister 2020; van den Hoek et al. 2022). Harnessing the full potential of the combined power of all these approaches should enable important strides forward towards the holy grail: the reconstitution of the entire process of PSI assembly in vitro from purified components. In addition, it will facilitate the rational design of modified PSI complexes that contribute to the development of novel sustainable energy technologies. For example, by rerouting the electrons arriving at the acceptor side of PSI with suitably designed in vitro and in vivo systems, molecular hydrogen (H_2_) can be produced from essentially unlimited sources (water and light; Nelson 2009; Redding et al. 2022). In this way, research on PSI can potentially inspire new solutions for solar energy conversion in biotechnology and synthetic biology (Janna Olmos and Kargul 2015; Teodor and Bruce 2020).

## Dive Curated Terms

The following phenotypic, genotypic, and functional terms are of significance to the work described in this paper:

ALB3 Gramene: AT2G28800
ALB3 Araport: AT2G28800

LHCA1 Gramene: AT3G54890
LHCA1 Araport: AT3G54890

ALB4 Gramene: AT1G24490
ALB4 Araport: AT1G24490

LHCA2 Gramene: AT3G61470
LHCA2 Araport: AT3G61470

LHCA3 Gramene: AT1G61520
LHCA3 Araport: AT1G61520

NFU3 Gramene: AT4G25910
NFU3 Araport: AT4G25910

ycf37 Gramene: slr0171
ycf37 Araport: slr0171

HCF101 Gramene: AT3G24430
HCF101 Araport: AT3G24430

PSAF Gramene: AT1G31330
PSAF Araport: AT1G31330

PSAG Gramene: AT1G55670
PSAG Araport: AT1G55670

PSAL Gramene: AT4G12800
PSAL Araport: AT4G12800

PSAK Gramene: AT1G30380
PSAK Araport: AT1G30380

LHCA4 Gramene: AT3G47470
LHCA4 Araport: AT3G47470

PPD1 Gramene: AT4G15510
PPD1 Araport: AT4G15510

PSAO Gramene: AT1G08380
PSAO Araport: AT1G08380

PSAN Gramene: AT5G64040
PSAN Araport: AT5G64040

PIR1 Gramene: Cre01.g014000
PIR1 Araport: Cre01.g014000

CGL71 Gramene: Cre12.g524300
CGL71 Araport: Cre12.g524300

Y3IP1 Gramene: AT5G44650
Y3IP1 Araport: AT5G44650

PSA3 Gramene: Cre03.g183400
PSA3 Araport: Cre03.g183400

PsaA Gramene: ATCG00350
PsaA Araport: ATCG00350

APO1 Gramene: AT1G64810
APO1 Araport: AT1G64810

ATAB2 Gramene: AT3G08010
ATAB2 Araport: AT3G08010

AtFKBP20-2 Gramene: AT3G60370
AtFKBP20-2 Araport: AT3G60370

cpSRP43 Gramene: AT2G47450
cpSRP43 Araport: AT2G47450

cpSRP54 Gramene: AT5G03940
cpSRP54 Araport: AT5G03940

FKB20-2 Gramene: Cre13.g577850
FKB20-2 Araport: Cre13.g577850

HCF145 Gramene: AT5G08720
HCF145 Araport: AT5G08720

LHCA5 Gramene: AT1G45474
LHCA5 Araport: AT1G45474

LHCA6 Gramene: AT1G19150
LHCA6 Araport: AT1G19150

NFU2 Gramene: AT5G49940
NFU2 Araport: AT5G49940

OTP51 Gramene: AT2G15820
OTP51 Araport: AT2G15820

PBF2 Gramene: AT3G42630
PBF2 Araport: AT3G42630

CEPA1/PBF8 Gramene: AT3G56010
CEPA1/PBF8 Araport: AT3G56010

PSA2 Gramene: AT2G34860
PSA2 Araport: AT2G34860

PsaB Gramene: ATCG00340
PsaB Araport: ATCG00340

PsaC Gramene: ATCG01060
PsaC Araport: ATCG01060

PSAD-1 Gramene: AT4G02770
PSAD-1 Araport: AT4G02770

PSAD-2 Gramene: AT1G03130
PSAD-2 Araport: AT1G03130

PSAE-1 Gramene: AT4G28750
PSAE-1 Araport: AT4G28750

PSAE-2 Gramene: AT2G20260
PSAE-2 Araport: AT2G20260

PSAH-1 Gramene: AT3G16140
PSAH-1 Araport: AT3G16140

PSAH-2 Gramene: AT1G52230
PSAH-2 Araport: AT1G52230

PsaI Gramene: ATCG00510
PsaI Araport: ATCG00510

PsaJ Gramene: ATCG00630
PsaJ Araport: ATCG00630

PSBP4 Gramene: Cre08.g362900
PSBP4 Araport: Cre08.g362900

PYG7 Gramene: AT1G22700
PYG7 Araport: AT1G22700

THA8 Gramene: AT3G27750
THA8 Araport: AT3G27750

Y3IP1/CGL59 Gramene: Cre06.g280650
Y3IP1/CGL59 Araport: Cre06.g280650

ycf3 Gramene: CreCp.g802301
ycf3 Araport: CreCp.g802301

ycf4 Gramene: CreCp.g802302
ycf4 Araport: CreCp.g802302

ZNJ1 Gramene: Cre11.g475850
ZNJ1 Araport: Cre11.g475850

ycf51 Gramene: sll1702
ycf51 Araport: sll1702
